# A step-wise, deterministic and fatal mouse model of myeloid neoplasm with spontaneous acquisition of patient-relevant RTK–RAS mutations

**DOI:** 10.1038/s41388-026-03964-w

**Published:** 2026-09-04

**Authors:** Marija A. Zarocsinceva, Moosa Qureshi, Nicola K. Wilson, George Giotopoulos, Blaise Musabe, Katherine H. M. Sturgess, M. S. Vijayabaskar, Sarah J. Kinston, Ryan Asby, David J. Adams, Brian J. P. Huntly, Fernando J. Calero-Nieto, Berthold Göttgens

**Affiliations:** 1 Cambridge Stem Cell Institute, Department of Haematology, University of Cambridge, Cambridge, UK; 2 Wellcome Sanger Institute, Wellcome Genome Campus, Hinxton, Cambridge, UK

**Keywords:** Leukaemia, Cancer genomics

## Abstract

Leukaemia arises through the stepwise transformation of healthy haematopoietic cells, yet the asymptomatic premalignant phase and its progression to overt disease remain poorly understood. To model this process, we engineered a patient-derived *CEBPA* mutation into Hoxb8-FL multipotent murine progenitors and transplanted them into syngeneic mice, capturing a clinically silent premalignant stage. All recipients developed overt disease after ~12 months with 100% penetrance and all acquired secondary RTK–RAS mutations, often with identical amino acid changes to those in patients. Single-cell transcriptomics and phenotypic profiling showed that premalignant mutant cells adopt a plasmacytoid dendritic progenitor–like state in vitro which generates both myeloid and B-lymphoid lineages during premalignancy in vivo, with individual tumours restricted to one lineage. The specificity for RTK-RAS mutations coupled with ongoing differentiation, reflects clinically relevant biological contexts thus providing a tractable model of myeloid neoplasm for mechanistic studies and drug discovery.

## Introduction

Cancer evolution often spans many years. In leukaemia, reconstructions from single-cell-derived genome sequences suggest that initial preleukaemic mutations in paediatric and adult cases can occur *in utero* [1, 2]. Stepwise acquisition of mutations increases fitness of individual preleukaemic clones, ultimately leading to the development of aggressive disease. While genomics can reconstruct tumour evolution retrospectively, the early stages of leukaemogenesis are typically inaccessible where individuals are asymptomatic. Deep sequencing of acute leukaemias has revealed that genetically diverse blast populations often derive from a single founding clone [3–5] and the original preleukaemic mutations are commonly found in relapse samples [5–7]. This suggests that rare preleukaemic cells evade cytotoxic chemotherapy yet retain the capacity to regenerate the disease. New experimental models to study cancer progression from its earliest stages are therefore essential to gain a comprehensive understanding of both initial tumour evolution, as well as to design interventions that target the disease at its root, thereby improving patient outcomes.

Retroviral transduction models have long been a mainstay of leukaemia modelling [8, 9], whereby a pool of mouse bone marrow (BM) stem/progenitor cells are transduced with a retrovirus carrying a leukaemia oncogene ex vivo, followed by transplantation to track disease development in in vivo. However, the substantial heterogeneity of the transduced progenitor cells obscures the precise identity of the initiating cell. To better control for the nature of the initiating cell state, we have previously taken advantage of conditionally immortalised Hoxb8-FL cells [10], which represent a in vitro counterpart of lymphoid-primed multipotent progenitors (LMPP) that can be maintained in culture by combining the known self-renewal properties of *Hox* genes with the pro-survival function of the Flt3 ligand. Of note, LMPP have been suggested as one possible candidate cell of origin for AML as well as a potential normal counterpart to LSCs [11]. Using the *MLL-ENL* leukaemia oncogene, we previously demonstrated how Hoxb8-FL cells provide a powerful cell system for in vivo leukaemia modelling, taking advantage of its homogeneity coupled with retained multilineage differentiation capacity [12].

The *CEBPA* gene is commonly mutated in Acute Myeloid Leukaemia (AML) patients, believed to be an early leukaemogenic event [13], relapse samples usually retain the *CEBPA* mutation from the initial tumour [14, 15], and germline *CEBPA* variants may affect development of AML, collectively highlighting early and disease-initiating roles for CEBPA. Of particular clinical relevance, mutations that disrupt the basic leucine-zipper (bZIP) region of CEBPA have been recognised as a distinct prognostic subgroup [16–18], mirrored by a mouse retroviral transplant study reporting that a bZIP *CEBPA*-N321D mutant caused the most rapid and aggressive disease [19]. Here we combined the Hoxb8-FL cellular model with a *CEBPA*-N321D retrovirus to capture the effects of *CEBPA* mutation on the haematopoietic system in a tightly controlled setting. We reveal how *CEBPA* N321D can substitute for the self-renewal function of Hoxb8 in vitro and generate a premalignant state when transplanted in vivo. Progression to aggressive disease in vivo entails a long latency period and requires the acquisition of secondary driver mutations, exquisitely targeting five members of RTK-RAS pathway. The new model thus captures all critical stages of leukaemogenesis, for which we report comprehensive mutation-, as well as single cell gene expression states. Our new model thus combines a well-defined cell of origin with deterministic evolution from preleukemic fully transformed leukaemic cells harbouring clinically relevant and somatically acquired RTK-RAS pathway mutations, all accessible for mechanistic and therapeutic experimentation in a syngeneic fully immunocompetent in vivo setting.

## Results

### N321D mutant *CEBPA* endows Hoxb8-FL cells with Hox-independent propagation

**Fig. 1:**
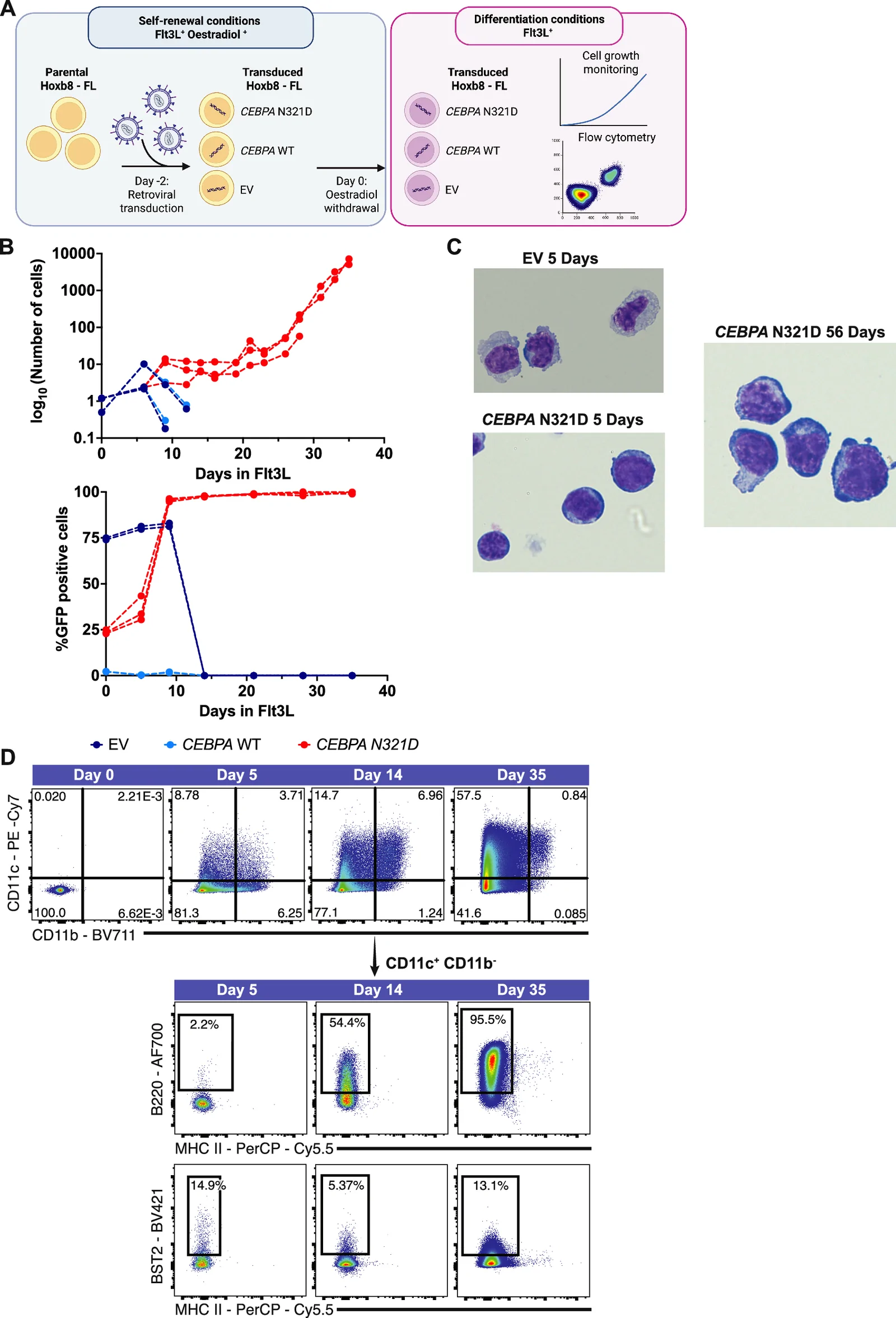
N321D mutant *CEBPA* endows Hoxb8-FL cells with Hox-independent propagation. **A** Schematic of establishment and characterisation of *CEBPA* N321D model. Hoxb8-FL cells were transduced with different retroviral constructs and subjected to β-oestradiol withdrawal (abrogating Hoxb8 signalling). Differentiation potential of the resulting cells was assessed by monitoring cell growth and characterising haematopoietic marker expression via flow cytometry. Created in BioRender.com Zarocsinceva, M. A. (2026) https://BioRender.com/ffeeaci. **B** Cell numbers and the percentage of GFP-positive cells over time for transduced cells cultured in Flt3L (differentiation conditions). The displayed values are individual values of *n* = 2 for EV and *CEBPA* WT and *n* = 3 for *CEBPA* N321D. **C** Brightfield images of Romanowsky-stained GFP^+^ EV and GFP^+^ *CEBPA* N321D cells cultured in Flt3L media for 5 days and of *CEBPA* N321D cells cultured in Flt3L media for 56 days. **D** Flow cytometry analysis of dendritic cell marker expression in *CEBPA* N321D cells in Flt3L media.

A common drawback of experimental models to study leukaemia initiation and progression is the phenotypic and functional heterogeneity of the starting population. Hoxb8-FL cells provide a relatively homogeneous pool of progenitors. Based on the recognised significance of *CEBPA* C-terminal bZIP mutations in leukaemia, we transduced multipotent Hoxb8-FL cells with retroviral MSCV-GFP constructs carrying either the mutant or wild-type *CEBPA* (*CEBPA* N321D and *CEBPA* WT, respectively), or an empty vector control (EV) (Fig. 1A). To allow time for retroviral integration, cells were expanded for two days in self-renewal conditions (β-oestradiol and Flt3L) but subsequently transferred to differentiation conditions lacking oestradiol (hereafter Flt3L). Only the *CEBPA* N321D mutant cells continued to proliferate throughout the duration of the experiment in Flt3L media in the absence of oestradiol, demonstrating *Hox*-independent self-renewal capacity driven by *CEBPA* N321D (Fig. 1B). EV and *CEBPA* WT cells by contrast differentiated, and remained viable for only 9 days consistent with the original Hoxb8-FL publication [10]. Notably, *CEBPA* WT-transduced cells showed a reduced proportion of GFP⁺ cells at early time points, likely reflecting rapid differentiation and loss of proliferative capacity under Hoxb8 self-renewal conditions.

### Hox-independent mutant *CEBPA* cells adopt a dendritic progenitor phenotype

At day 5 post β-oestradiol withdrawal, EV cells displayed classic dendritic cell features including lateralised reniform nuclei, vacuolised cytoplasm and a few dendritic projections (Fig. 1C), which were absent in the *CEBPA* N321D cells analysed at the same time point. After 56 days in Flt3L, a subset of *CEBPA* N321D cells showed partial dendritic features but did not reach mature morphology.

Redecke et al. [10] showed that Hoxb8-FL cells grown in the presence of Flt3L differentiate into a mixed population of conventional dendritic cells (CD11c^+^, CD11b^+^, MHC II^+^, B220^-^ cDCs) and plasmacytoid dendritic cells (defined by CD11c^+^, CD11b^-^, B220^+^ pDCs). Our *CEBPA* N321D cells showed an increase in CD11c^+^ CD11b^-^ over time (Fig. 1D), progressively becoming more positive for the pDC marker B220. *CEBPA* N321D cells remained MHC II^-^ throughout the experiment and only lowly expressed BST2, suggestive of a stalled progenitor-like state (Supplementary Fig. 1A–C). Of note, *CEBPA* WT transduced cells differentiated upon transduction even with Hox active (Supplementary Fig. 1A, 1B), consistent with prior reports that forced CEBPA induces differentiation of 32Dcl3 cells even under self-renewal conditions [20] and more generally can reprogramme chromatin and repress cell-cycle/self-renewal pathways to restore differentiation in proliferative or leukaemic cells [21–25]. RNA-sequencing of four independent batches of *CEBPA* N321D cells at sequential time points during Flt3L culture showed highly consistent growth rates, levels of CD11c and B220 expression as well as transcriptomic homogeneity, thereby confirming high reproducibility of generating the Hox-independent *CEBPA* N321D self-renewing phenotype (Supplementary Fig. 2A–C). Collectively, these results demonstrate that *CEBPA* N321D cells have a defect in DC differentiation and instead acquire a self-renewing pDC progenitor-like phenotype.

### *CEBPA* N321D cells give rise to a long-latency myeloid neoplasm in vivo

**Fig. 2:**
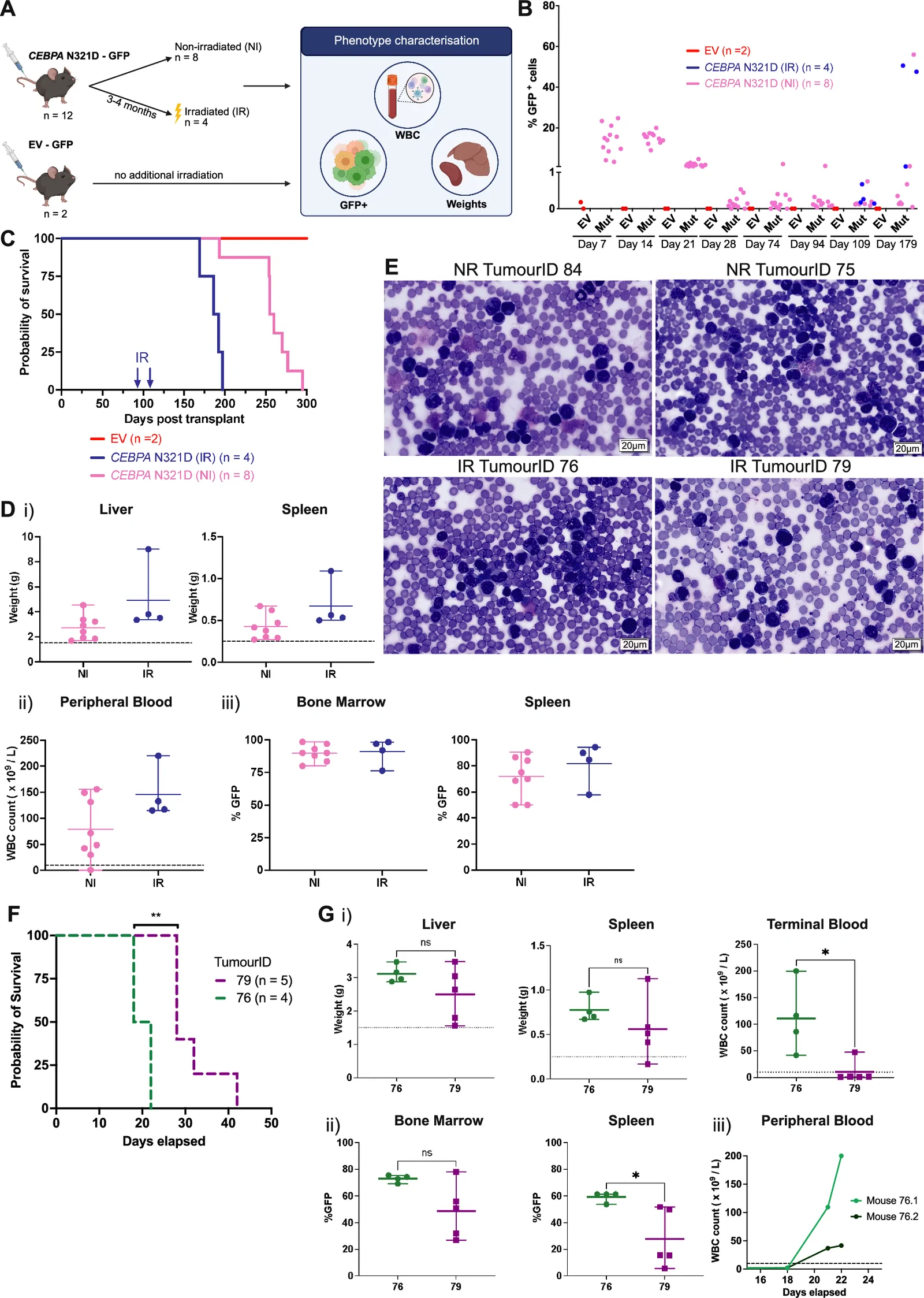
*CEBPA* N321D cells give rise to a long latency leukaemia in vivo. **A** Schematic of mouse transplantation experiments to assess the leukaemogenic potential of the *CEBPA* N321D cells. Lethally irradiated mice were injected with either *CEBPA* N321D cells (*n* = 12) or EV cells (*n* = 2). Mice injected with *CEBPA* N321D cells were subdivided into two cohorts: Cohort non-irradiated, NI (*n* = 8) received no additional doses of irradiation. Irradiated cohort, IR (*n* = 4) received an additional low dose of (mutagenic) irradiation (1 Gy) 3 or 4 months after transplantation. Disease progression was assessed by measuring WBC and %GFP positive cells in the peripheral blood. Symptomatic mice were culled, and their liver and spleen were weighed. Created with BioRender.com Zarocsinceva, M.A. (2026) https://BioRender.com/b60q012. **B** Peripheral blood chimerism of mice injected with *CEBPA* N321D or EV cells. **C** Survival of primary recipients. **D** Characterisation of the disease phenotype. i) Graphs show hepatomegaly and splenomegaly in symptomatic mice, ii) terminal WBC, iii)%GFP in BM and %GFP in spleen. **E** H&E-stained peripheral blood smears from four symptomatic mice injected with *CEBPA* N321D cells. **F** Survival curves of secondary recipients transplanted with BM cells from two primary tumours (Mantel-Cox test, **p value = 0.004). **G** Characterisation of secondary disease phenotype. Unless otherwise stated, significance calculated with Mann–Whitney test. i) Liver and spleen weights at time of disease presentation (ns *p*-values 0.413 and 0.190). Terminal WBC of symptomatic mice (*p-value = 0.032). ii) GFP% in BM and spleen (*p*-values 0.190 and 0.016). iii) Time course of WBCs of two mice injected with cells from Tumour ID 76. Dashed lines in (**D**) and (**G**) indicate upper limits of normal liver weight (1.5 g), spleen weight (0.25 g) and WBC (×10^9^/L).

To validate if *CEBPA* N321D cells had leukaemogenic potential in vivo, we transplanted GFP + *CEBPA* N321D or EV cells together with supportive BM cells into lethally irradiated recipients (Fig. 2A). Transplantation of *CEBPA* WT cells was not feasible, as they underwent differentiation in Hox active media, consistent with our earlier observations. GFP^+^ cells were detected in all recipients 7 days post-transplantation (Fig. 2B), albeit with higher chimerism for *CEBPA* mutant (>10%) than EV cells (<1%). No EV cells were detected past day 7. This short-term repopulating capacity of EV cells is consistent with the original Hoxb8 study [10], which showed that endogenous oestrogen levels in recipient mice are insufficient to sustain Hoxb8 activity in vivo. GFP+ cells were detected in *CEBPA* N321D recipients at multiple sampled time points, with occasional absences likely reflecting detection limit constraints given the small peripheral blood volumes sampled, as evidenced by the reappearance of GFP+ cells at later time points. Long-term presence of GFP positive cells in the peripheral blood demonstrated that a subset of *CEBPA* N321D cells displayed sustained self-renewal activity in vivo and produced output detectable in peripheral blood, with the remainder likely differentiating or failing to home to the BM. Sustained engraftment without rapid disease onset suggested that secondary mutations were likely required for disease progression. To test this hypothesis, 4 of the 12 mice transplanted were subjected to an additional low dose (1 Gy) of ionising irradiation (Irradiated, IR), as a further mutagenic stimulus, 3–4 months post the initial transplantation (Fig. 2A).

All mice injected with *CEBPA* N321D cells ultimately developed leukaemia, whereas no mice injected with control EV cells showed any signs of leukaemia development (Fig. 2C). The non-irradiated (NI) cohort had a median survival of 257 days, whereas recipients from the IR cohort developed disease around 190 days post transplantation. All symptomatic mice showed splenomegaly and hepatomegaly and elevated white blood counts (WBC), consistent with the phenotypic presentation of leukaemia (Fig. 2Di, ii, Supplementary Fig. 3A). Flow cytometry showed GFP-positive cells in the BM (>75%) and spleen (>50%) of all animals, confirming that leukaemia was initiated by mutant *CEBPA* N321D cells (Figure 2Diii).

Peripheral blood smears demonstrated increased numbers of myeloid cells, with blast-like cells detectable in the majority of symptomatic animals (Fig. 2E and Supplementary Fig. 3B). To determine whether BM cells from diseased animals possess tumour-initiating capacity and self-renewal potential, two primary tumours (Tumour ID 76 and 79) were transplanted into a new cohort of mice. All animals developed disease 18 to 42 days post-transplantation. Of note, a marked difference in disease latency was seen between the recipients of each tumour, potentially reflecting differences in their secondary driver mutations and/or leukaemic stem cell numbers (Fig. 2F). Secondary recipients had increased liver and spleen weights, increased WBCs (all recipients of tumour 76, but not 79) (Fig. 2Gi) and infiltration of GFP-positive cells in BM and spleen (Fig. 2Gii). Highlighting the potential utility of our model for future mechanistic and therapeutic studies, we observed an increase in GFP^+^ cells as an early indicator of disease progression (Fig. 2Giii). While these secondary transplantations were performed using tumours derived from irradiated recipients, we did not observe differences in mutational or phenotypic features between tumours arising in irradiated versus non-irradiated settings, suggesting comparable engraftment potential. In addition, extramedullary infiltration was observed in the liver, lung, and kidney in recipients of both tumours, with variable extent. Despite differences in disease latency and peripheral blood counts, no major morphological or immunophenotypic differences were observed between tumours #76 and #79 (Supplementary Fig. 4).

### Leukaemia onset requires secondary mutations in the RTK-RAS pathway

**Fig. 3:**
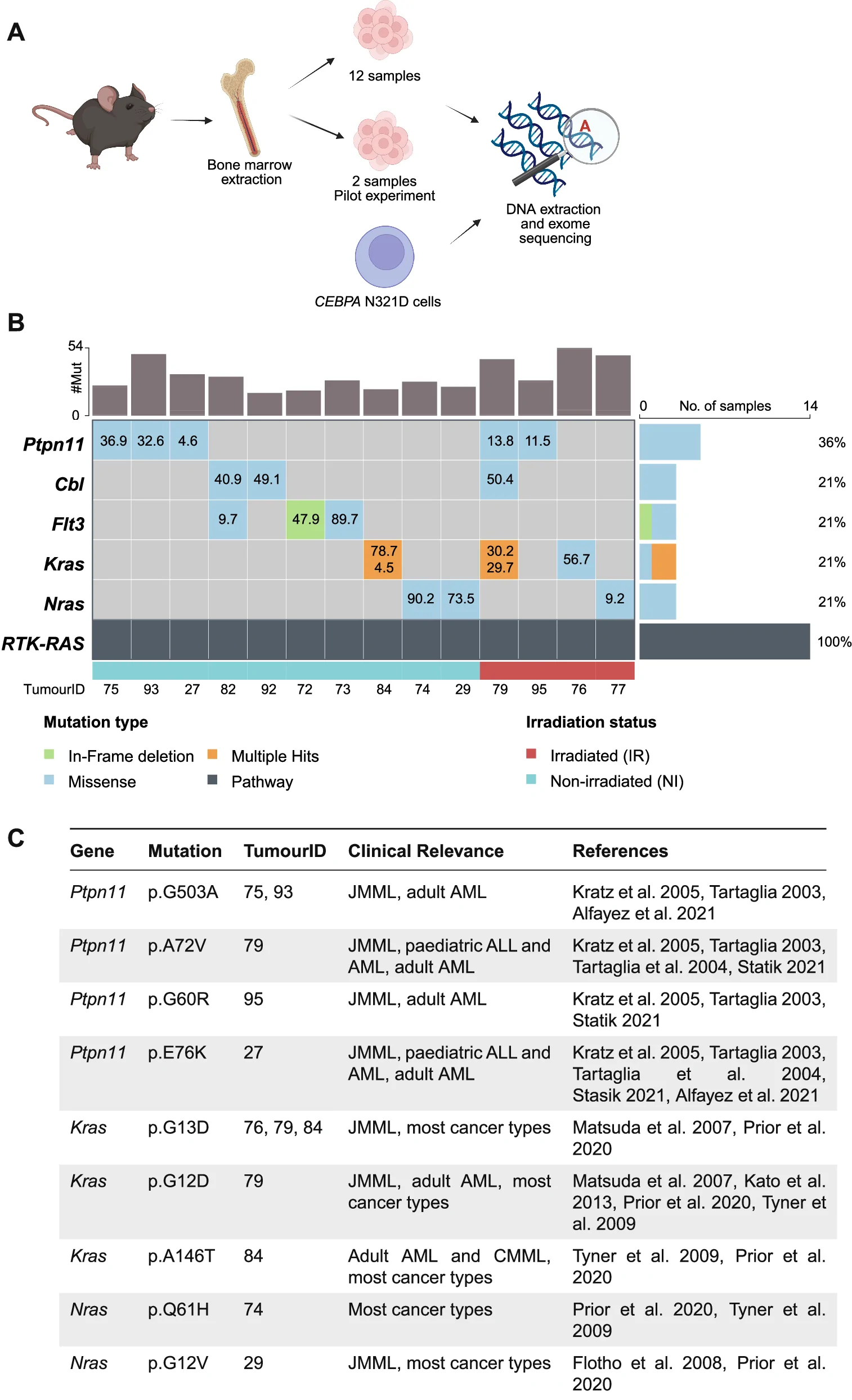
Leukaemia onset requires secondary mutations in the RTK-RAS pathway. **A** Diagram showing the outline of the exome sequencing experiment. Created with BioRender.com Zarocsinceva, M. A. (2026) https://BioRender.com/za5iwxr. **B** Mutations found in the genes of the RTK-RAS pathway. The bar chart on top of the plot shows the number of mutations found in each of the tumour samples. The bar chart on the right depicts the % of samples with mutations found in each of the 5 genes. The calculated VAF is displayed inside the box. If more than one mutation was found in a gene, the VAFs for each of the mutations are displayed together in the same box. **C** Mutations found in this study that are frequently found in haematological malignancies [27–36].

The long latency of disease following transplantation of *CEBPA* N321D cells, and the observed acceleration of disease progression with mutagenic stress prompted us to exome sequence DNA from 12 primary tumours, the pre-injection *CEBPA* N321D cell line (as a reference) and 2 tumour samples from a small pilot transplantation experiment (Fig. 3A). We identified a median value of 28 mutations per sample, with the IR samples showing slightly elevated numbers of mutations, as expected. However, despite their elevated mutational burden no mutations were specific to the IR cohort (compared to the NI samples) (Supplementary Figs. 5A, B), suggesting that the *CEBPA* N321D may exert a strong selective pressure on the secondary drivers.

The most overrepresented pathway was the RTK-RAS pathway (present in 14/14 samples), which included mutations in *Ptpn11*, *Cbl*, *Flt3*, *Kras* and *Nras* (Fig. 3B). Several samples contained multiple mutations in RTK-RAS pathway genes, although most had only a single mutation. The RTK-RAS pathway is central for cell growth, differentiation, and cell survival (Supplementary Fig. 6A), with pro-oncogenic mutations found across a wide spectrum of human cancers [26, 27]. RTK-RAS mutations presented with high variant allele frequency (VAF), reaching >50% VAF in some samples hinting at the potential presence of homozygous clones. Moreover, RTK-RAS pathway mutations were among the highest calculated VAFs across all the mutations (Supplementary Fig. 6B) and were commonly found within key functional domains of the respective protein products (Supplementary Fig. 6C). RTK-RAS pathway mutations therefore represent pivotal drivers of leukaemogenic progression in our model of CEBPA-mutated myeloid neoplasm.

Strikingly, most mutations in RTK-RAS proteins recapitulated the exact mutations found in human patients (Fig. 3C), including adult Acute Myeloid Leukaemia (AML) [28–30], paediatric AML and Acute Lymphoblastic Leukaemia (ALL) [31] and Juvenile Myelomonocytic Leukaemia (JMML) [32–36]. 98% of *RAS* mutations in human cancers are found at three amino acid residues: G12, G13 or Q61. We observed *KRAS* G12D, *KRAS* G13D, *NRAS* G12V and *NRAS* Q61H mutations, all of which have been reported as common *RAS* mutations across a variety of human cancers (Catalogue of Somatic Mutations in Cancer, reviewed in Muñoz-Maldonado et al. [37]). Exome-sequencing analysis therefore confirmed that the *CEBPA* N321D mutation is not sufficient to drive overt leukaemia in our model, yet imposes a strong selection for de novo acquisition of clinically relevant RTK-RAS pathway mutations.

### Leukaemic transformation is a late event during disease progression

**Fig. 4:**
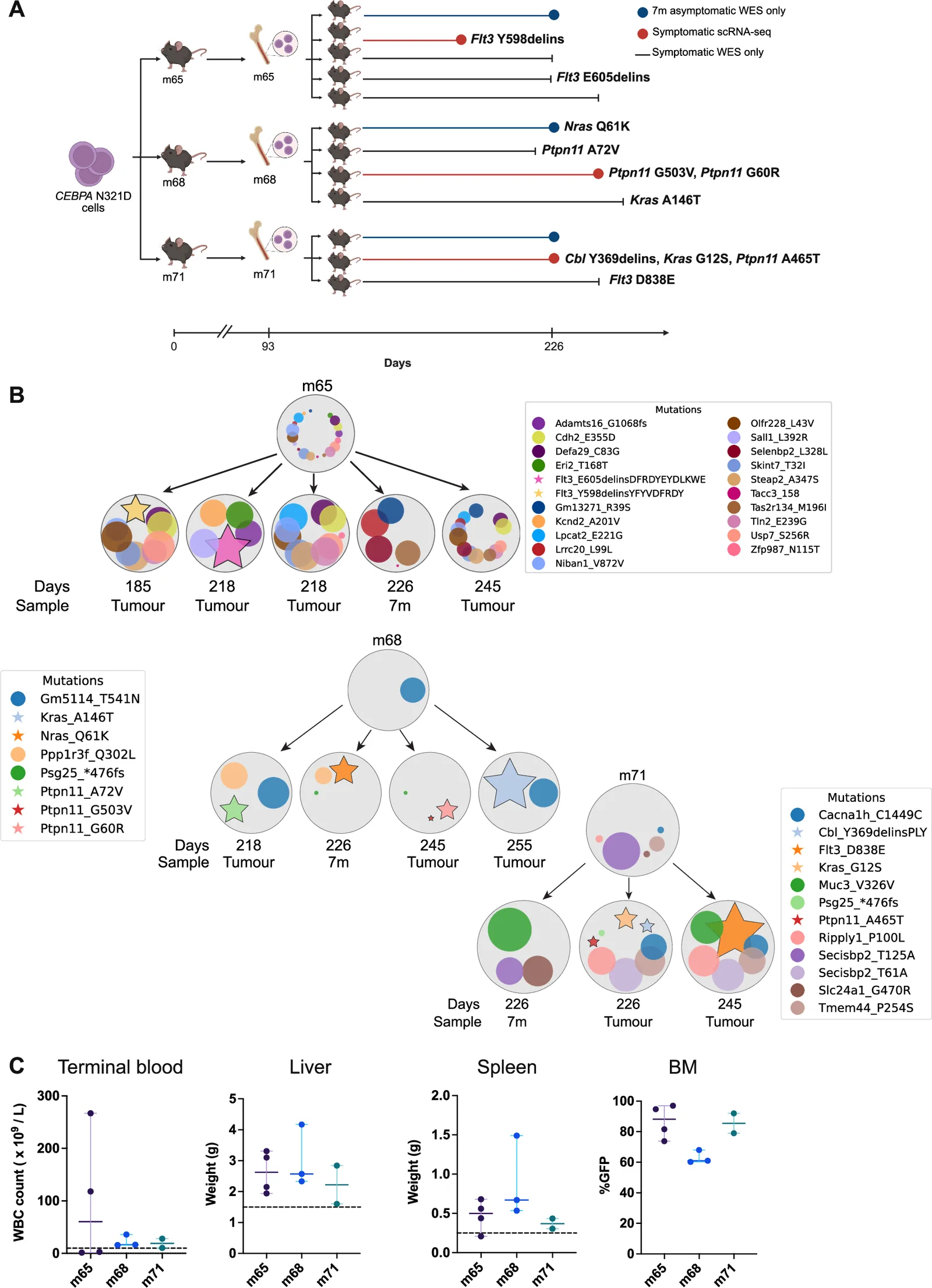
Leukaemia transformation is a late event during disease progression. **A** Schematic of the experiment to determine the timeline of *CEBPA* N321D-mediated tumorigenesis. *CEBPA* N321D cells were injected into three mice (m65, m71 and m68). After 93 days, these mice were culled, and their BM cells transplanted into secondary recipients. One mouse from each of the three groups was culled at 226 days post primary transplantation. The remaining animals were monitored for leukaemia development. The timeline at the bottom of the figure represents the survival duration of the secondary recipients, with RTK-RAS mutations identified in their BM cells annotated alongside the survival curves. Each sample is labelled with the number of days after the primary transplantation. BM cells from all samples were analysed by WES. Symptomatic samples submitted for 10x Genomics single-cell RNA-seq are highlighted in red. Created with BioRender.com Zarocsinceva, M. A. (2026) https://BioRender.com/zcsolr6. **B** Schematic of mutations found in the BM cells of the 93-day donor and secondary recipients. Each passenger mutation is displayed as a circle and a RTK-RAS driver is shown as a star shape. The size of the shape is proportional to the VAF of that mutation. Dominant clones within each sample can be identified by the largest symbols, allowing comparison of clonal contributions across related samples. To simplify the schematic, we filtered out mutations that appeared at high VAFs in all samples. Additionally, for the m65 samples, only mutations that emerged from the 93-day donor sample onward are included. All shared mutations and their corresponding VAFs can be found in Supplementary Fig. 7. **C** Graphs show hepatomegaly and splenomegaly, increased WBCs in terminal bloods and high contribution of GFP-positive cells in symptomatic animals.

We next investigated the temporal emergence of the RTK-RAS pathway mutations. To differentiate between early- versus late-acquisition, *CEBPA* N321D cells were injected into murine recipients and three mice (m65, m68 and m71) were culled 3 months post-transplantation and their BM cells harvested. An aliquot of unfractionated BM cells was retained for subsequent molecular analysis, with the remainder transplanted into secondary recipients (Fig. 4A) from each primary case (‘donor cells’) transplanted into at least three secondary recipients. One secondary recipient from each group was culled 226 days after the primary transplantation (133 days post-secondary transplantation) as an intermediate sample (thereafter called ‘7 months intermediate’) whilst the remaining mice were left for tumour development.

Tumours developed asynchronously amongst the recipients that received cells from the same donor at early time points (Fig. 4A). Moreover, RAS-RTK mutations were only detected in the symptomatic mice, with none detected in the 3-month donor samples. One single 7-month mouse from the m68 donor group had no clinical signs of disease, but had a *Nras* Q61K mutation (VAF 53.3%) and a 33% GFP-positive population in the BM, compared to only 5% in samples lacking driver mutations. Therefore, we suggest that this sample captured an early transformation event with malignant clone expansion but prior to overt leukaemia symptoms. Finally, all tumours displayed different driver mutations, suggesting independent mutation acquisition within the reconstituted marrow in the donor, yet interestingly the mutations were in the same five members of the RAS-RTK pathway as seen in previous experiments: *Flt3*, *Nras*, *Ptpn11*, *Kras* and *Cbl* (Fig. 4B). Three mice developed tumours with the exact amino acid changes found from the first transplantation experiment, namely *Ptpn11* A72V, *Ptpn11* G60R and *Kras* A146T, demonstrating remarkable specificity to fully transform our model.

Disease was confirmed in all symptomatic secondary recipients based on a combination of features, including increased GFP⁺ cells in BM, splenomegaly and hepatomegaly, with leukocytosis observed in a subset of animals. (Fig. 4C). We also catalogued non-recurrent neutral passenger mutations that accumulated over time, as barcodes to infer the evolution of different clones within our samples, which identified distinct clonal expansions in the individual mice from all three groups of secondary recipients (Fig. 4B, Supplementary Fig. 7), confirming that leukaemic transformation occurred independently. For example, two tumours that arose from m65 cells not only acquired distinct mutations in the *Flt3* gene but also exhibited the expansion of a different subset of passenger mutations. Similarly, a mutation in the *Gm5114* gene present in the 3-month donor cells for the m68 group, showed increased VAF in two tumours, yet was absent in the other two samples. Moreover, each secondary recipient acquired a unique mutation in the RTK-RAS pathway, underscoring independent clonal evolution. Finally, although two tumour samples from the m71 group shared four passenger mutations, the 7-month intermediate did not display these mutations and showed an expansion of two other passenger mutations from the 3-month sample. Collectively, these results are consistent with a model whereby multiple clones exist by the three-month time point, each with the potential to subsequently evolve into a tumorigenic clone.

### Overt leukaemia develops without a differentiation block where individual tumours produce either lymphoid or myeloid output

To further characterise the *CEBPA* N321D cells before and after transformation, we performed single-cell RNA-sequencing (scRNA-Seq) on the same BM aliquots used for exome-sequencing (Fig. 4A). To focus the scRNA-Seq on the *CEBPA* N321D-derived cells, we processed sorted GFP^+^ cells from all samples. We analysed (i) *CEBPA* N321D cells collected on the day of primary transplantation (“Preinjected” - day 0 Fig. 4), (ii) cells from the m65, m68, and m71 mice at 93 days post-injection (“Asymptomatic”), and (iii) cells obtained from the secondary transplant recipients of the m65, m68, and m71 BM donor cells as the recipients were being sacrificed because of their full-blown leukaemia (“Symptomatic” - highlighted by red circles in Fig. 4A). Additionally, we profiled GFP^-^ host progenitor cells, defined here as Lineage^-^ cKit^+^, from BM samples collected from the 93-day group (m65, m68, m71) to have a reference set of progenitors obtained from the same mice on the same day.

**Fig. 5:**
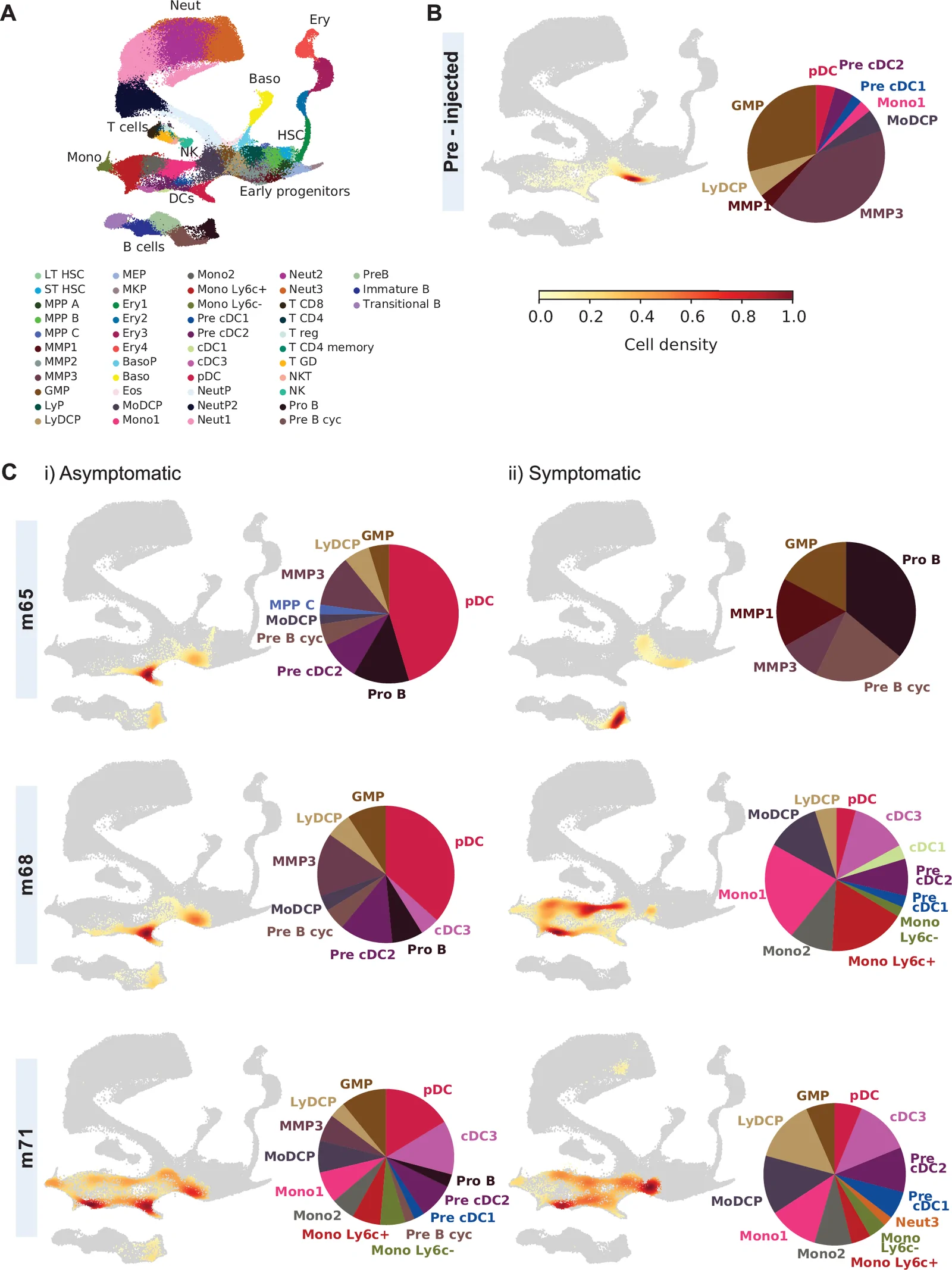
Leukaemogenesis in the *CEBPA* N321D model depends on the secondary mutation. **A** Non-diseased mouse BM atlas was used as a reference for projection and cell type annotation of our samples. LT HSC Long term haematopoietic stem cell, ST HSC short term haematopoietic stem cell, MPP multipotent progenitor, MMP myeloid-monocyte progenitor GMP granulocyte-monocyte progenitor, LyP lymphoid progenitor, LyDCP lymphoid dendritic cell progenitors, MEP megakaryocyte-erythroid progenitors, MKP megakaryocyte progenitor, Ery erythrocyte, BasoP basophil progenitor, Baso basophil, Eos eosinophil, MoDCP monocyte-dendritic cell progenitors, Mono monocyte, cDC conventional dendritic cell, pDC plasmacytoid dendritic cell, Neut neutrophil, NK natural killer cell. **B** Results of cell type annotation of *CEBPA* N321D cells collected on the day of transplantation, ‘pre-injected’; **C** Cell type annotation of i) Cells collected 93 days post primary transplantation from mice m65, m68 and m71 (asymptomatic); ii) symptomatic recipients of either the m65, m68 or m71 donor cells.

Evolution of cellular states during leukaemia development was assessed by projecting the new single cell transcriptomes onto a non-diseased mouse whole BM atlas (Sturgess et al., in preparation), comprised of 196,986 cells isolated from the BM of non-diseased wild-type mice combining haematopoietic stem/progenitor enriched populations (lineage^-^ c-kit^+^ and lineage^-^ c-kit^+^ Sca1^+^) with total mononuclear BM cells thus providing a comprehensive reference for non-diseased mouse haematopoiesis (Fig. 5A). The 93-day host progenitor cells matched across a broad range of haematopoietic progenitor populations (Supplementary Fig. 8). The ‘pre-injected’ *CEBPA* N321D cells most closely resemble immature monocytes, particularly myeloid-monocyte progenitors (MMP3) and granulocyte-monocyte progenitor (GMP) populations (Fig. 5B). Three months (93 days) after transplantation (Fig. 5Ci), cells harvested from three individual recipient mice (m65, m68, and m71) appear highly similar to one another mapping across a broad range of cell populations, with a notable expansion of pDC-like cells and the emergence of lymphoid-like, immature B-cell populations.

The two symptomatic tumours derived from m68 and m71 donors mapped to immature myeloid progenitors and monocyte populations, whereas the tumour derived from the m65 donor mapped to immature lymphoid progenitors and B-lymphoid cells (Fig. 5Cii). Cross referencing with the exome sequencing data outlined in the previous section showed that the m68 tumour contained two mutations in *Ptpn11*, the m71 tumour carried a different *Ptpn11* mutation along with additional mutations in *Cbl* and *Kras*, whereas the m65 tumour harboured a *Flt3* mutation. Immunophenotypic analysis of all tumours in this study confirmed both myeloid- and lymphoid-biased disease, with 7/21 tumours exhibiting a mixed/lymphoid-like phenotype. (Supplementary Fig. 9). Notably, no clear correlation was observed between specific RTK–RAS mutations and lineage phenotype, suggesting that mutation identity alone does not dictate myeloid versus lymphoid disease output.

Single-cell RNA-Seq thus showed that progression from preleukaemia to full-blown disease in this model is not associated with a strict differentiation block and accumulation of cells, since both stages are characterised by a differentiation axis going from immature to more mature cells. Moreover, overt leukaemia can adopt either a myeloid or lymphoid phenotype resulting in a somewhat perplexing situation whereby the model displays exquisite specificity for a particular pathway (RAS-RTK) for secondary mutations yet permits the development of leukaemias of different lineage phenotypes. Of note, both these features are reminiscent of Juvenile myelomonocytic leukaemia (JMML).

## Discussion

Understanding the mechanisms of oncogenic progression remains a major challenge in cancer biology, largely due to the difficulty of accessing the early stages of the disease in human patients. Given the parallels between relapse and the transition from premalignant to full-blown cancer, a deeper understanding of leukaemogenic mechanisms therefore has major implications both for relapse prevention and treatment of established disease. In this study, we describe a novel tractable model of leukaemogenesis with characteristic acquisition of secondary RTK-RAS pathway mutations, that can be used to disentangle molecular and phenotypic cancer evolution in vivo.

Primary transplantation experiments were performed in lethally irradiated recipients, which is the standard approach to ensure efficient donor cell engraftment in haematopoietic transplantation models. Unlike conventional transplantation-based mouse models that use heterogeneous progenitor cells as their starting population, we took advantage of the Hoxb8-FL cell system that allows introduction of oncogenes into a well-defined progenitor population, which maintains primary cell characteristics upon transplantation. We demonstrate that the C-terminal *CEBPA* N321D mutation removes the requirement for Hoxb8 for in vitro culture, leading to a pool of self-renewing progenitors with a pDC-like phenotype that can be propagated indefinitely with the supplementation of Flt3L. Transplantation of these differentiation-stalled cells in vivo showed them to be pre-malignant, requiring specific secondary mutations to develop full-blown disease with a latency of almost a year.

A particularly striking feature of the resulting disease was the exquisite specificity of secondary mutations, with all tumours analysed acquiring RTK-RAS pathway secondary mutations. This observation aligns with prior human and murine work showing that activating RTK–RAS pathway alterations commonly act as secondary/cooperating events in myeloid leukaemogenesis, whereby proliferative drivers such as FLT3 and RAS are frequently acquired late during progression, and that oncogenic RAS often requires a permissive pre-existing mutational background [6, 38–40]. Moreover, while mutation order can vary between patients and models, functional data support a model in which transcription-factor lesions create a permissive state for later signalling hits, for example mutant CEBPA occurring before mutant CSF3R to initiate AML [13].

Many of the RTK-RAS mutations acquired in the endogenous mouse gene loci resulted in the exact same amino acid changes seen in human patients, including some of the most common RAS mutations found across human cancers. Mutations in *Kras*, *Nras*, *Ptpn11* and *Cbl* genes serve as one of a constellation of diagnostic markers for JMML, a rare but aggressive paediatric myeloid neoplasm, characterised by overactive RAS signalling [41]. Other alterations in RTK‑RAS pathway genes, such as *FLT3-*mutations, have also been described, albeit at a lower frequency [42, 43]. *CEBPA* N321D preleukaemic cells demonstrated pDC-like characteristics. CEBPA is a key regulator of myeloid and dendritic cell differentiation, and the Hoxb8-FL system is cultured with Flt3L, a cytokine known to promote dendritic cell development. Therefore, the emergence of pDC-like populations in this model likely reflects the underlying differentiation potential of the *CEBPA*-mutant progenitor cells under these conditions. Single-cell transcriptomics showed that pDC-like populations were more prominent in asymptomatic mice, suggesting that they may represent an early cell population that becomes restricted during leukaemic progression as additional cooperating mutations are acquired. These observations highlight the dynamic differentiation landscape of *CEBPA*-mutant cells during disease evolution in this model. Human malignancies associated with pDC defects include the rare disease entities blastic plasmacytoid dendritic cell neoplasm (BPDCN) and AML with pDC expansion (pDC-AML), and neither have been particularly reported to associate with RAS-specific mutational profiles. Of note, DC-like lineage capacity of JMML cells has previously been described [44–46]. Another distinguishing feature of the leukaemias observed in this study is that they lacked the differentiation block commonly associated with myeloid malignancies such as AML. JMML is likewise characterised by a low percentage of blasts (<20%) [47, 48] and the presence of differentiating cells [49, 50], thus lacking the differentiation block commonly observed in acute leukaemias [47]. Furthermore, our results showed that both myeloid and B-lymphoid output was produced during the preleukaemic phase, whereas full-blown leukaemias produced output for just one of those downstream lineages. In rare case reports, individual JMML patients have been described in the clinic, with an abnormal B-cell population [51] and disease progression to a pre-B cell acute lymphoblastic leukaemia [52]. Setting it apart from other mouse models, our RTK‑RAS–driven model shows high specificity for secondary mutations, yet flexibility in terms of haematopoietic lineage output, highlighting similarities to selected features observed in JMML while also underscoring important biological differences. In particular, our model differs from JMML in several key aspects, including the presence of an initiating CEBPA mutation and the late acquisition of RTK–RAS pathway mutations, and is therefore not intended to represent a direct model of JMML but instead captures selected overlapping features of RAS-driven myeloid malignancies.

RAS proteins had been long thought of as undruggable although recent efforts have resulted in several KRAS inhibitors currently in Pre-clinical or Phase I clinical studies (reviewed in [53, 54]). Tumour-derived BM cells from our new model readily engraft into secondary recipients thus permitting in vivo testing of any new therapeutics, including the development of drug resistance. In addition, we were able to derive stable cell lines from primary tumours, which require distinct culture conditions compared to preleukaemic *CEBPA* mutant cells, providing a complementary in vitro platform for future functional and drug sensitivity studies. Unlike transgenic models, the mutations arise within the endogenous gene locus. Mutant RAS is therefore under the control of endogenous regulatory sequences, which is ideal to model resistance mechanisms driven by altering gene expression including epigenetic processes. Moreover, our model overcomes a key limitation of patient-derived xenograft models (PDX), where patient-derived cells are transplanted into immunocompromised animals that have been genetically engineered to permanently lack an immune system. This not only causes the loss of a functional tumour microenvironment that is often essential in evaluating drug responsiveness, but also makes it impractical to assess any combination therapies where new RAS inhibitors are combined with the rapidly growing array of immune-oncology therapeutics.

Although primary human tissues provide a valuable tool for studies into carcinogenesis, material is often limited. Moreover, in vitro cell culture from such samples can be extremely challenging, especially in malignancies that are rare and lack a full differentiation block, including JMML [55]. Here we report a new model of leukaemogenesis that provides access to the full timeline of tumour development, thus enabling direct comparisons across all stages of malignant transformation, to not only develop hypotheses for subsequent mechanistic investigation, but also to test the efficacy of new therapeutic approaches. With regards to the latter, our model stands out by being fully immunocompetent and developing patient-relevant RAS-RTK mutations within the endogenous gene loci, thus providing a robust preclinical platform to accelerate the development of precision therapies, bridging critical gaps between mechanistic discovery and clinical translation.

## Methods

Detailed methods are provided in the Supplementary files.

### Cell lines and culture conditions

Hoxb8-FL and B16-FLT3 cell lines were kindly provided by the Hans Häcker laboratory and grown as described [10]. Flt3L conditioned medium constituted supernatant from B16-FLT3 cell, seeded in 10 cm dishes and grown at 37 °C and 5% CO_2_ in RPMI 1640 medium (Sigma R8758) supplemented with 10% FBS (Gibco 10270-106), 0.1% 2-Mercaptoethanol, 1% Penicillin/Streptomycin (Sigma, P0781), 1% Glutamine (Sigma, G7513) for 2–3 days until confluent.

293T cells were cultured in Dulbecco’s Modified Eagle Medium (DMEM) supplemented with 10% FBS, 1% Penicillin/Streptomycin (Sigma, P0781) and 1% Glutamine (Sigma, G7513). Cells were cultured in 100 mm/150 mm dishes at 37 °C and 5% CO_2_. Cells were passaged every 2–3 days with Trypsin-EDTA (Sigma, T3924) and kept at 30–90% confluency.

Differentiation media refers to the Flt3L media that induced differentiation of Hoxb8-FL cells as described [10] and consisted of the same components that the Hoxb8-FL media except without the addition of β -oestradiol.

### Retroviral constructs

Retroviral vectors encoding human CEBPA-WT, CEBPA-N321D, or empty vector (EV) were generated by cloning cDNAs into pMSCV-IRES-eGFP using Gibson assembly. Full strategy is described in the Supplementary Methods.

### Retroviral production and transduction

Viral supernatants were produced by transient transfection of 293T cells and used to transduce Hoxb8-FL progenitors in the presence of polybrene. Transduced cells were cultured in self-renewing conditions for two days prior to differentiation assays.

### Differentiation assay

Hoxb8-FL cells transduced with EV, CEBPA WT, or CEBPA N321D were transferred to Flt3L-containing medium without β-oestradiol. Cultures were monitored by flow cytometry for expression of haematopoietic markers at multiple timepoints.

### Flow cytometry and cell sorting

Cells were stained with lineage-specific antibodies and viability dye and analysed on a BD LSR Fortessa (BD Biosciences). Cells were sorted by the CIMR Flow Core Facility using the Influx Cell Sorter (BD Biosciences). Compensation, antibody panels, and gating strategies are provided in Supplementary Methods.

### Microscopy

Cell morphology was assessed by cytospins stained with Rapid Romanowsky Stain Pack (TCS Biosciences, HS705). Microscopic images were acquired on a Zeiss Axio Imager.Z2 using Zen Pro software. Tissue sections (blood, spleen, liver, BM) were H&E stained by the Cambridge Stem Cell Institute Histology facility and imaged with OLYMPUS VS200 Slideviewer.

### Single-cell and bulk sequencing

Cells were FACS-sorted for single-cell RNA seq using 10x Genomics Chromium (v3.1 chemistry) with sequencing on an Illumina NovaSeq. DNA was extracted from cells and subjected to whole exome sequencing (WES) on Illumina NovaSeq platforms by Dr. David Adams’ laboratory at the Wellcome Trust Sanger Institute or by Novogene (UK). For low-cell RNA-seq, Smart-seq2 protocols were adapted with minor modifications as before [56–58]. Library preparation, QC, and sequencing parameters are described in Supplementary Methods.

### Animals and in vivo transplantation

C57BL/6J mice were housed in pathogen-free in individually ventilated cages under a 12:12 h light–dark cycle, with food and water available ad libitum. Experiments were conducted under a UK Home Office project license (registration number: PP3042348), as per UK Home Office regulations, and reviewed by the University of Cambridge Animal Welfare Ethical Review Body. For transplantation assays, cells were injected into lethally irradiated CD45.2 recipients, together with CD45.1/2 helper cells. Primary tumour formation, secondary transplants, and serial time-course experiments were performed as outlined in Supplementary Methods. Disease progression was monitored by blood counts and flow cytometry.

### Bioinformatics

Sequencing reads were aligned to a custom mm10 reference genome augmented with pMSCV, GFP, and *CEBPA* N321D sequences. Single-cell RNA-seq data were processed with 10x Genomics Cell Ranger Software Suite (version 7.0.1) and analysed using Scanpy [59] and CellProject (https://github.com/Iwo-K/cellproject). WES reads were processed with BWA [60] and GATK [61], and variants annotated with ANNOVAR [62]. Smart-seq2 RNA-seq data were aligned with STAR, gene counts quantified with featureCounts, and downstream analysis performed using the in-house smqpp package (https://github.com/SharonWang/smqpp). Detailed parameter choices, filtering thresholds, and software versions are provided in Supplementary Methods.

### Figure generation

The authors would like to acknowledge that Figs. 1A, 2A, 3A, 4A and Supplementary Fig. 6A, C were created with BioRender.com.

## Supplementary information

Supplementary Figures and Methods

## Data Availability

Files are available in GEO https://www.ncbi.nlm.nih.gov/geo/query/acc.cgi?acc=GSE300817.

## Code availability

This study did not generate original code; example analysis notebooks: https://github.com/MariaZaro/StepwiseMyeloidModel/.
