# Supplementary material for: A step-wise, deterministic and fatal mouse model of myeloid neoplasm with spontaneous acquisition of patient-relevant RTK–RAS mutations
Source: Oncogene. 2026 Sep 4;45(42):4551–63. doi: 10.1038/s41388-026-03964-w (PMC13661246; doi:10.1038/s41388-026-03964-w)
Supplement: Supplementary file 1 [file 41388_2026_3964_MOESM1_ESM.pdf]

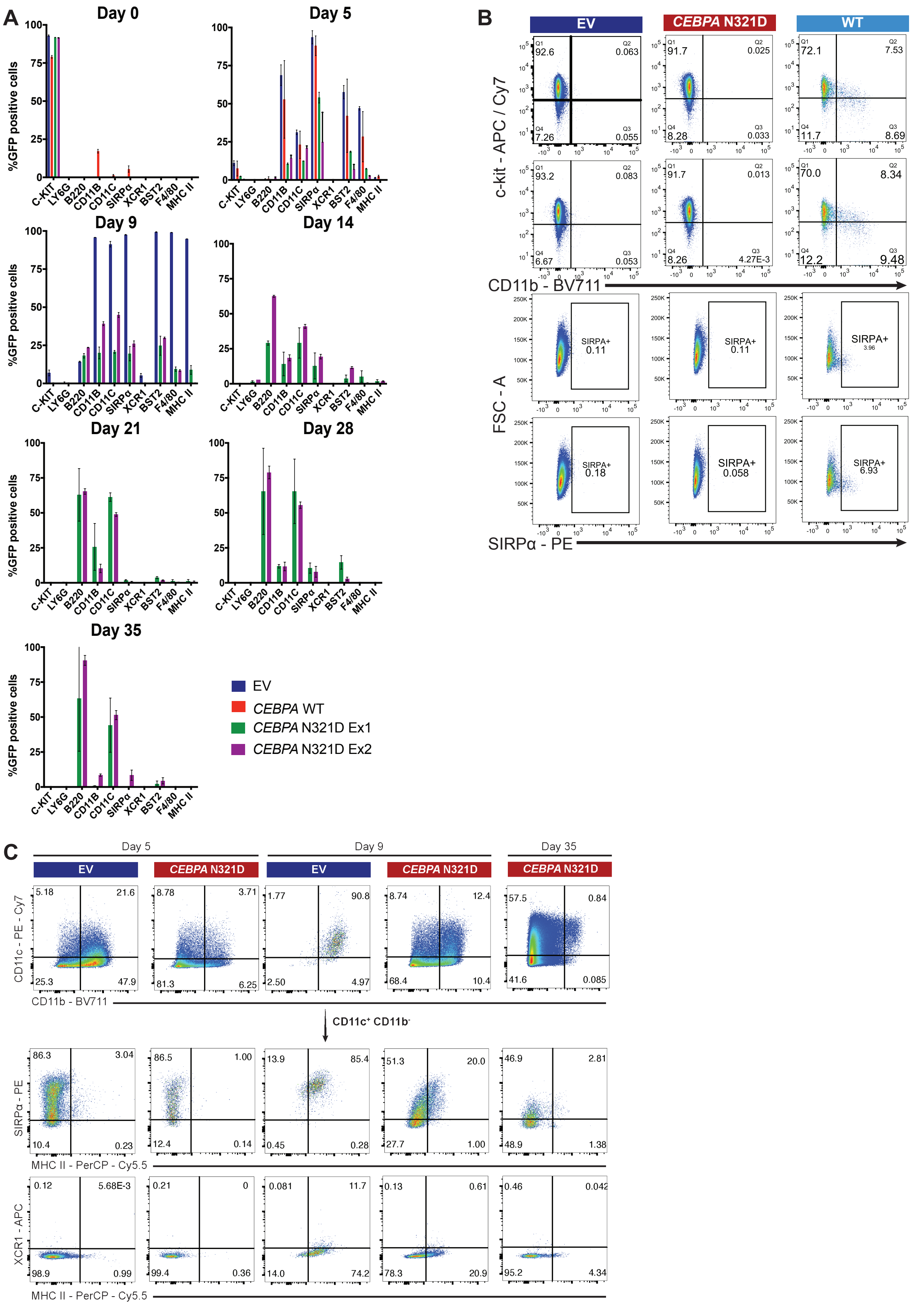

A
Day 0
Day 5
Day 9
Day 14
Day 21
Day 28
Day 35
%GFP positive cells
C-KIT
LY6G
B220
CD11B
CD11C
SIRPα
XCR1
BST2
F4/80
MHC II
EV
CEBPA WT
CEBPA N321D Ex1
CEBPA N321D Ex2
B
EV
CEBPA N321D
WT
c-kit - APC / Cy7
CD11b - BV711
FSC - A
SIRPα - PE
SIRPA+
C
Day 5
Day 9
Day 35
EV
CEBPA N321D
CD11c - PE - Cy7
CD11b - BV711
CD11c+ CD11b-
SIRPα - PE
MHC II - PerCP - Cy5.5
XCR1 - APC
MHC II - PerCP - Cy5.5

Supplementary Figure 1. A. Expression of all sampled haematopoietic markers in *CEBPA* WT, EV and *CEBPA* N321D cells. Two independent experiments of *CEBPA* N321D cells generation are included (Ex1 and Ex2 respectively). Each bar plot shows the calculated mean of two technical replicates and error bars represent the standard deviation. EV, *CEBPA* WT and *CEBPA* N321D Ex1 were performed in the same experiment and *CEBPA* N321D Ex2 repeated in a separate experiment. **B.** Expression of c-kit, CD11b and SIRPα in cells transduced with different retroviral vectors two days post transduction in oestradiol supplemented media. *CEBPA* WT cells show a more differentiated phenotype compared to EV and CEBPA N321D transduced cells. **C.** Expression of cDC haematopoietic markers in EV and *CEBPA* N321D transduced Hoxb8-FL cells cultured in Flt3L media over time (days). A small number of *CEBPA* N321D cells expressed both CD11c and CD11b at day 5 and day 9 in Flt3L conditions but this population was almost absent at day 35. This contrasts with the EV cells that showed a much higher CD11c^{+} CD11b^{+} population at day 5 and day 9. Interestingly, EV cells gave rise to the SIRPa^{+} cDC2 subtype but were unable to differentiate into the XCR1^{+} cDC1 subtype. There was only a very small number of SIRPa^{+} MHC II^{+} cells in the *CEBPA* N321D CD11c^{+} CD11b^{+} population present in long-term culture.

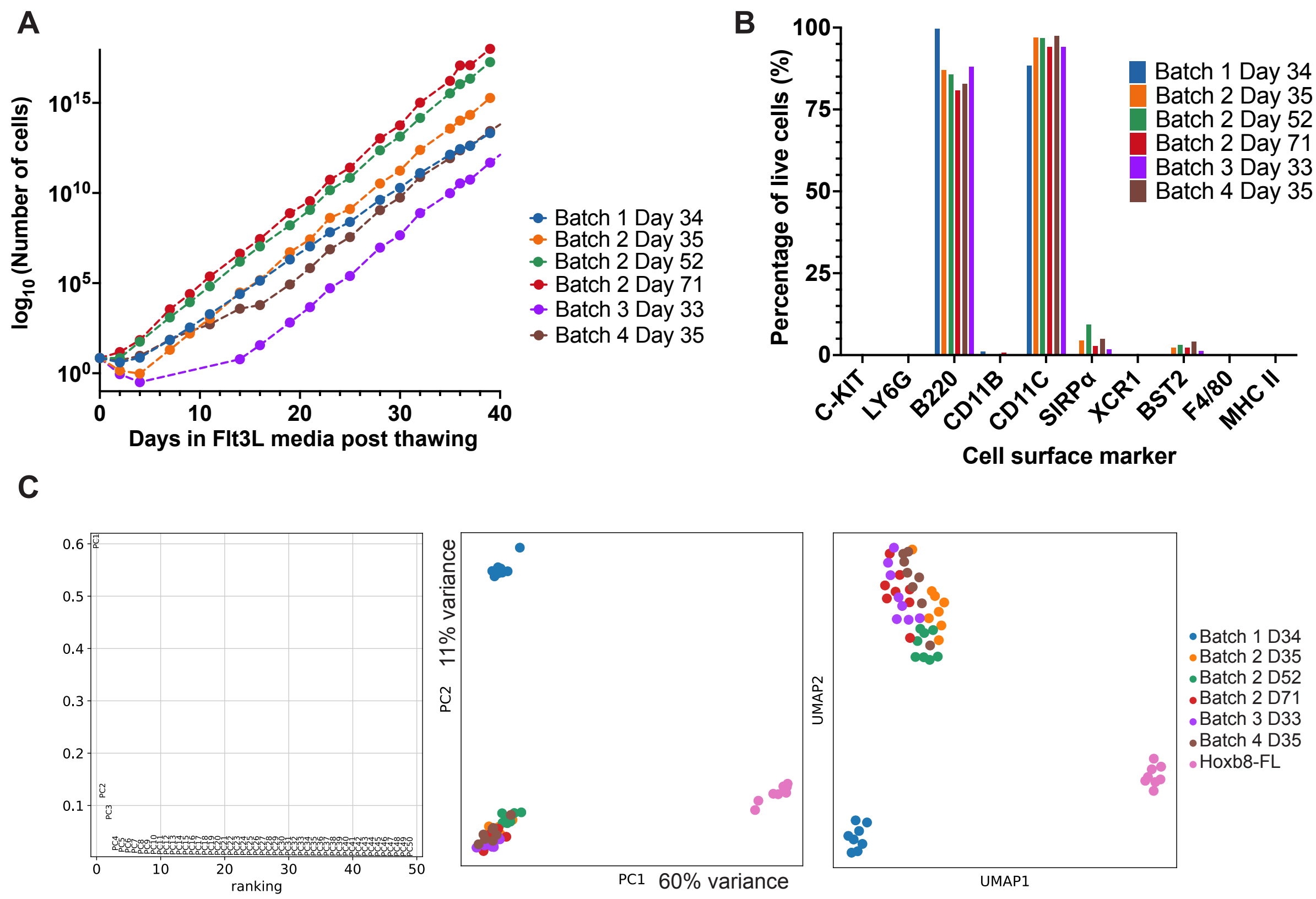

Supplementary Figure 2. A. Growth curves of independent replicates of *CEBPA* N321D cells grown in Flt3L media. **B.** Expression of haematopoietic markers as determined by flow cytometry analysis. Cells were collected after 38 days in Flt3L media post thawing. All samples are B220^{+} CD11C^{+}. A very small number of cells also showed expression of SIRPα and BST2 markers, apart from cells from Batch 1 day 34. **C.** Left: variance explained by each of the calculated PCs; middle: PCA plot showing PC1 and PC2; right: UMAP representation of the data coloured by batch. D - indicates number of days cultured in Flt3L media post thawing.

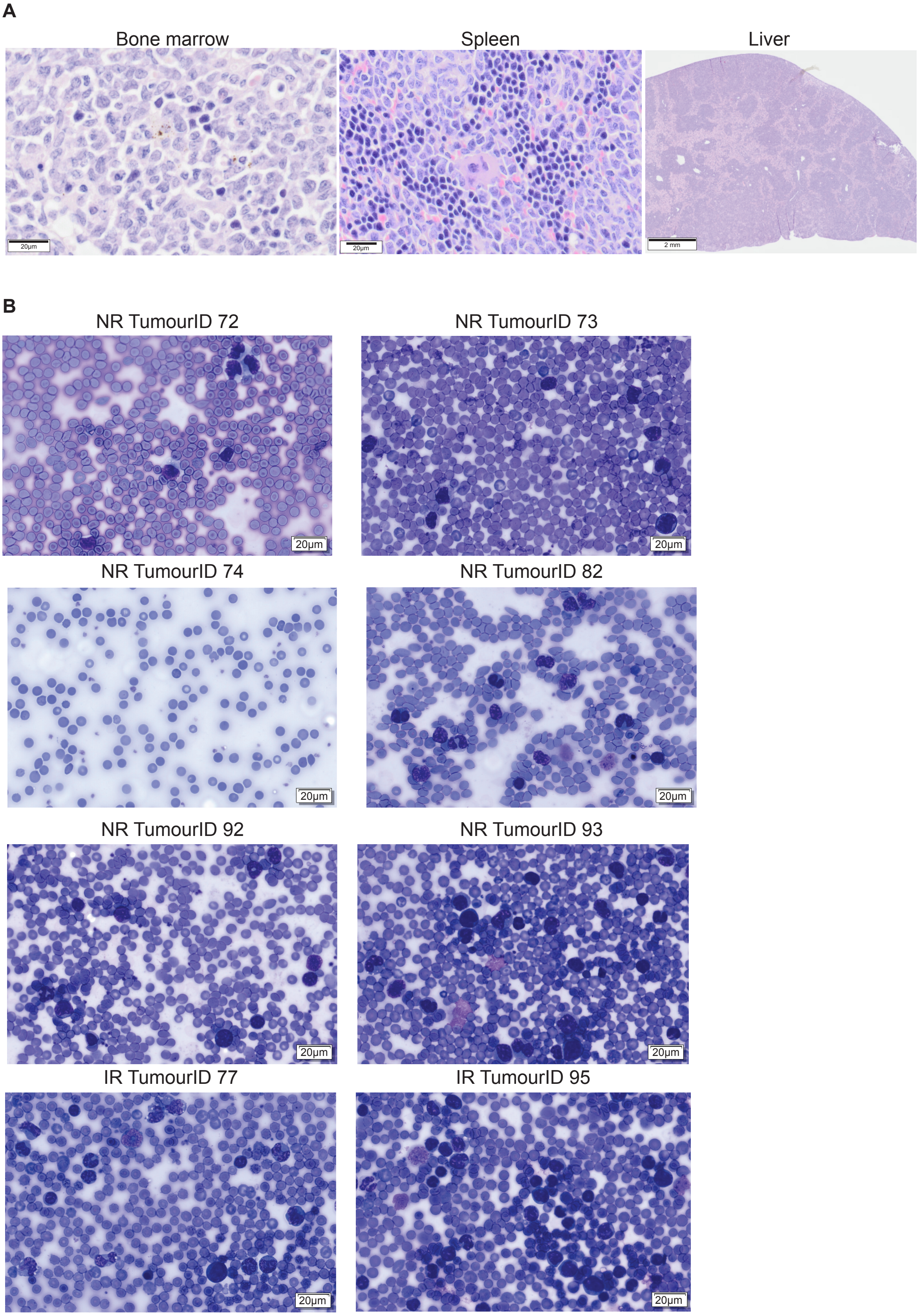
A
Bone marrow
Spleen
Liver
20µm
20µm
2 mm
B
NR TumourID 72
NR TumourID 73
NR TumourID 74
NR TumourID 82
NR TumourID 92
NR TumourID 93
IR TumourID 77
IR TumourID 95
20µm

Supplementary Figure 3. A. H&E-stained representative histology sections of liver, spleen and bone marrow from a mouse (Tumour ID 76) transplanted with the *CEBPA* N321D cells. **B.** H&E-stained peripheral blood smears from symptomatic mice injected with *CEBPA* N321D cells.

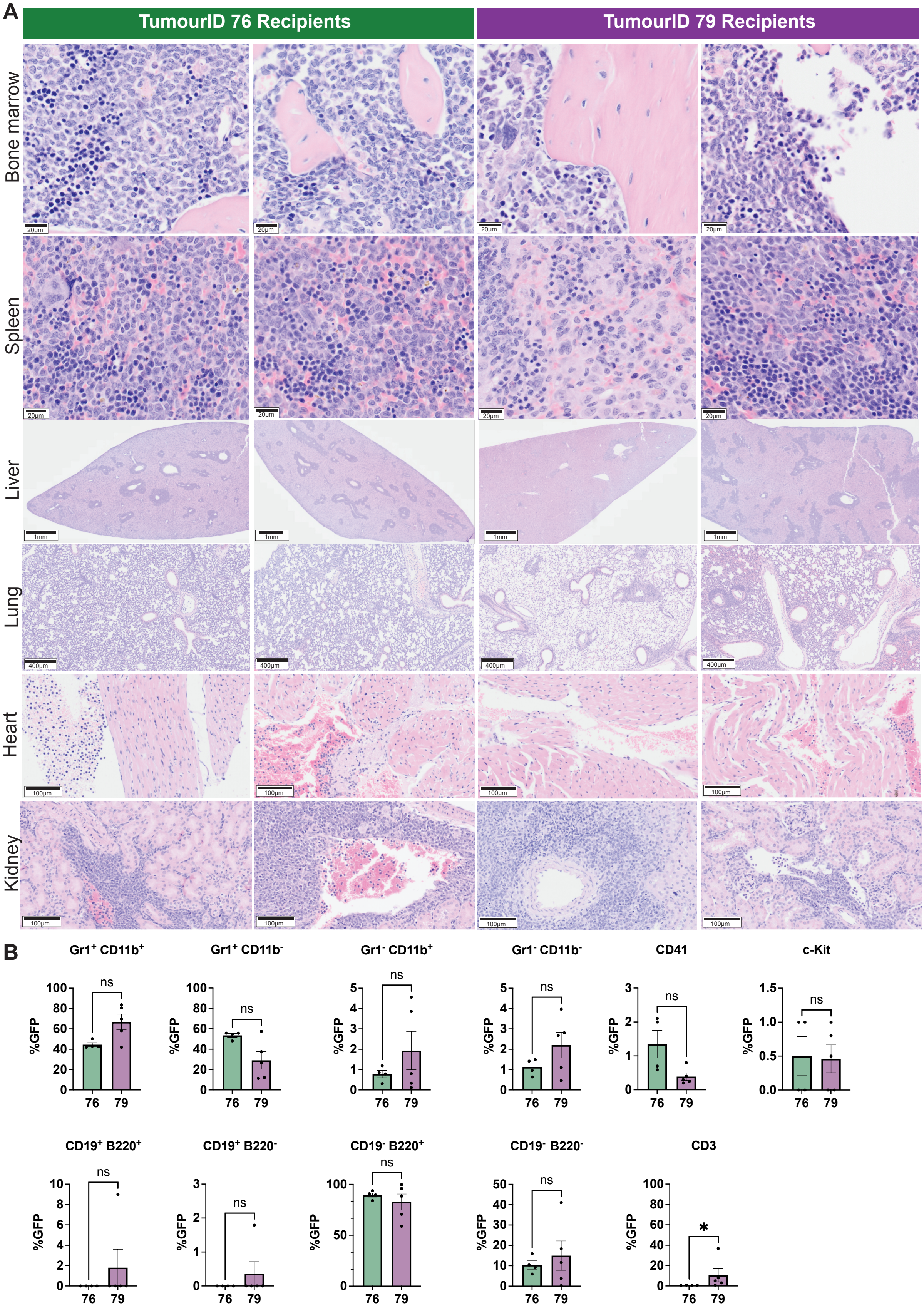

A
TumourID 76 Recipients
TumourID 79 Recipients
Bone marrow
Spleen
Liver
Lung
Heart
Kidney
B
Gr1+ CD11b+
Gr1+ CD11b-
Gr1- CD11b+
Gr1- CD11b-
CD41
c-Kit
CD19+ B220+
CD19+ B220-
CD19- B220+
CD19- B220-
CD3
%GFP
ns
*
76
79

Supplementary Figure 4. Histopathological and flow cytometric characterisation of secondary recipients of tumours #76 and #79. **A.** Representative H&E-stained sections of bone marrow, spleen, liver, lung, heart and kidney from recipients of tumours #76 and #79 (n = 2 cases per tumour). **B.** Quantification of cellular markers by flow cytometry in all recipients of tumours #76 and #79. Statistical significance was assessed using Mann–Whitney test (*$p < 0.05$).

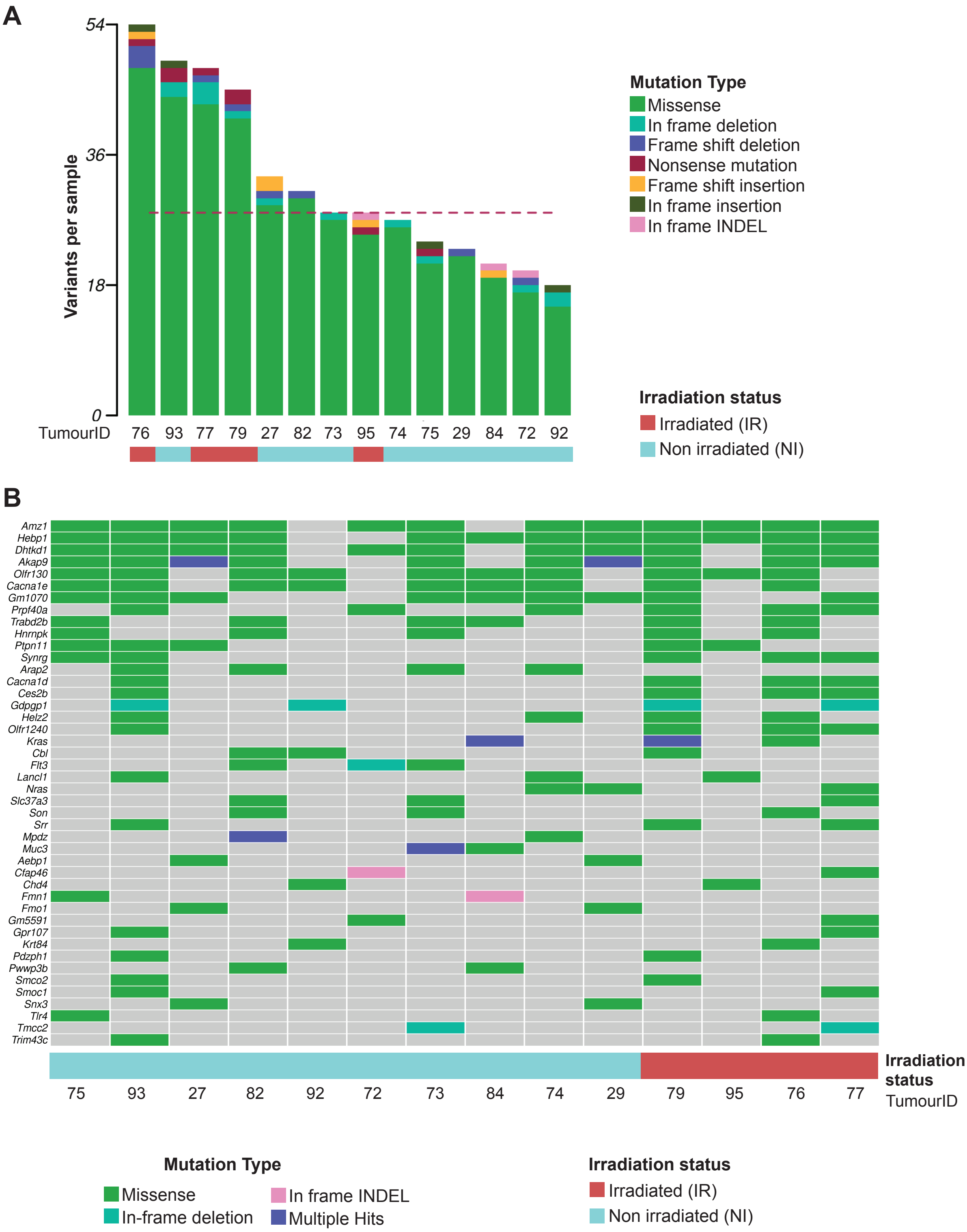

A
54
36
18
0
Variants per sample
TumourID
76 93 77 79 27 82 73 95 74 75 29 84 72 92
Mutation Type
Missense
In frame deletion
Frame shift deletion
Nonsense mutation
Frame shift insertion
In frame insertion
In frame INDEL
Irradiation status
Irradiated (IR)
Non irradiated (NI)
B
Amz1
Hebp1
Dhtkd1
Akap9
Olfr130
Cacna1e
Gm1070
Prpf40a
Trabd2b
Hnrnpk
Ptpn11
Synrg
Arap2
Cacna1d
Ces2b
Gdpgp1
Helz2
Olfr1240
Kras
Cbl
Flt3
Lancl1
Nras
Slc37a3
Son
Srr
Mpdz
Muc3
Aebp1
Cfap46
Chd4
Fmn1
Fmo1
Gm5591
Gpr107
Krt84
Pdzph1
Pwwp3b
Smco2
Smoc1
Snx3
Tlr4
Tmcc2
Trim43c
Irradiation status
75 93 27 82 92 72 73 84 74 29 79 95 76 77
TumourID
Mutation Type
Missense
In-frame deletion
In frame INDEL
Multiple Hits
Irradiation status
Irradiated (IR)
Non irradiated (NI)

Supplementary Figure 5. Mutations found in the *CEBPA* N321D BM cells. A. Bar chart showing the number and type of the acquired mutations in each of the 14 tumour samples. Irradiated samples Tumour ID 76, 77, 79 and 95 are marked in red. **B.** Oncoplot depicts mutations found in more than 2 samples. The presence of a mutation in a gene is shown by a non-grey colour, with the colour corresponding to a specific type of mutation. The genes are listed in order from highest to lowest based on the number of samples in which they are found to be mutated.

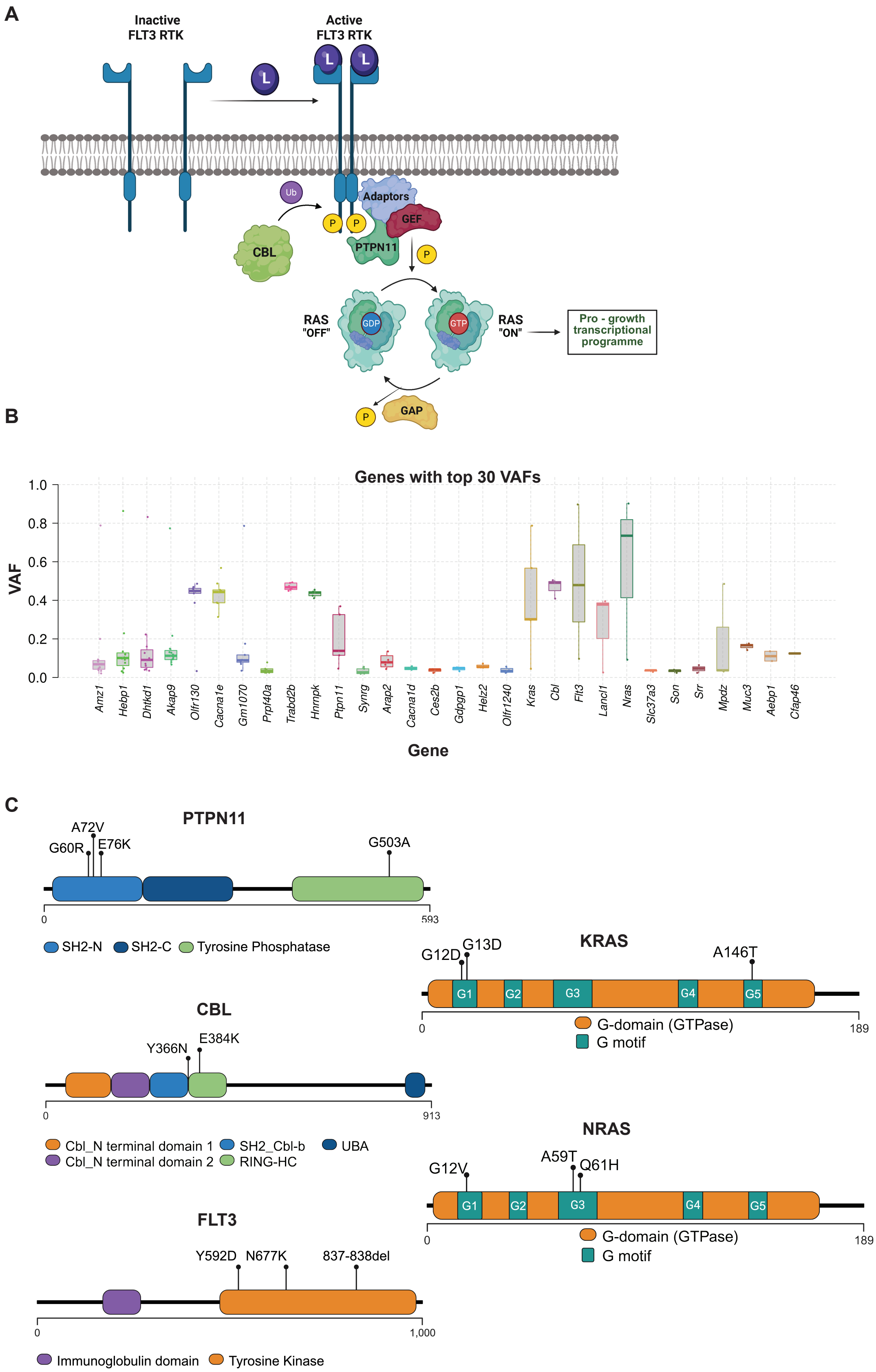
A
Inactive FLT3 RTK
Active FLT3 RTK
L
Ub
P
Adaptors
GEF
PTPN11
CBL
RAS "OFF"
GDP
GTP
RAS "ON"
Pro - growth transcriptional programme
GAP
B
Genes with top 30 VAFs
VAF
1.0
0.8
0.6
0.4
0.2
0.0
Amz1
Hebp1
Dhtkd1
Akap9
Olfr130
Cacna1e
Gm1070
Prpf40a
Trabd2b
Hnrnpk
Ptpn11
Synrg
Arap2
Cacna1d
Ces2b
Gdpgp1
Helz2
Olfr1240
Kras
Cbl
Flt3
Lancl1
Nras
Slc37a3
Son
Srr
Mpdz
Muc3
Aebp1
Cfap46
Gene
C
PTPN11
A72V
G60R
E76K
G503A
0
593
SH2-N
SH2-C
Tyrosine Phosphatase
KRAS
G12D
G13D
A146T
G1
G2
G3
G4
G5
0
189
G-domain (GTPase)
G motif
CBL
Y366N
E384K
0
913
Cbl_N terminal domain 1
SH2_Cbl-b
UBA
Cbl_N terminal domain 2
RING-HC
NRAS
G12V
A59T
Q61H
G1
G2
G3
G4
G5
0
189
G-domain (GTPase)
G motif
FLT3
Y592D
N677K
837-838del
0
1,000
Immunoglobulin domain
Tyrosine Kinase

Supplementary Figure 6. RTK-RAS pathway. A. RAS proteins are GTPases and cycle between "on"/"off" states. In the "off" state the protein is bound by GDP and in the "on" state by GTP that contains an extra phosphate group. Abbreviations: L, Ligand; Ub, Ubiquitin; P, Phosphate group. Created with BioRender.com Zarocsinceva, M. A. (2026) https://BioRender.com/qwvqse5. **B.** Variant allele frequency (VAF) for the top 30 most frequently mutated genes in *CEBPA* N321D tumours, arranged from most common to least common. VAF is calculated as the percentage of reads that do not correspond to the reference variant divided by total reads mapping to the region in a sample. **C.** Mutations in the RTK-RAS pathway are in functional protein domains that resemble those found in human disease. Lollipop plots of mutations found in the RTK-RAS pathway genes in the *CEBPA* N321D transformed tumours. The location of the mutations is shown alongside the functional domains of the proteins. Created with BioRender.com Zarocsinceva, M. A. (2026) https://BioRender.com/e5h1wpo.

m65

Mutation	93 / 3m	226 / 7m	218 / T	218 / T	185 / T	245 / T
Gm5114_R545R	42.5	50.0	56.7	51.2	63.5	41.4
Zfp987_N115T	9.8			10.3		
Eri2_T168T	8.1		48.7			
Kcnd2_A201V	5.1		48.5			
Krt86_A118A		50.7				27.9
Mid1ip1_G23G		49.3				25.9
Padi6_K326K		50.0				31.6
Pappa_P1409L				49.2	46.6	
Adamts16_G1068fs	11.9		47.2			
Tbx2_R345C		62.5				25.0
Sall1_L392R	6.1		52.1			
Tacc3_158	3.0	3.1				
Selenbp2_L328L	4.0	48.1				25.7
Gm13271_R39S	4.4	47.2				25.1
Lpcat2_E221G	16.8			50.4		21.7
Tas2r134_M196I	5.3	44.8				21.7
Lrrc20_L99L	7.3	50.0				17.8
Steap2_A347S	14.7			50.6	52.4	27.2
Cdh2_E355D	14.3			47.9	53.5	28.6
Gm5114_T541N		41.0	50.0	44.7	31.5	
Olfr228_L43V	19.1			49.2	55.0	31.9
Skint7_T32I	18.8			50.4	44.2	28.0
Tln2_E239G	17.9			51.1	45.1	25.7
Usp7_S256R	17.6			47.4	55.9	21.2
Defa29_C83G	16.8			46.6	47.3	26.3
Niban1_V872V	22.6			44.7	42.3	26.8
Flt3_Y598delinsYFYVDFRDY					56.2	
Flt3_E605delinsDFRDYEYDLKWE			88.3			

m68

Mutation	93 / 3m	226 / 7m	245 / T	218 / T	255 / T
Gm5114_R545R	47.3	40.8	52.6	45.8	46.1
Olfr730_V50I	23.2	47.4	42.2	44.0	53.8
Gm5114_T541N	43.6			54.8	50.0
Ppp1r3f_Q302L		29.4		46.7	
Psg25_*476fs		6.4	5.9		
Kras_A146T					96.9
Nras_Q61K		53.3			
Ptpn11_A72V				52.3	
Ptpn11_G503V			6.7		
Ptpn11_G60R			39.2		

m71

Mutation	93 / 3m	226 / 7m	226 / T	245 / T
Olfr127_L26V	46.1	52.6	48.2	49.0
Slc24a1_G470R	9.4	46.5		
Secisbp2_T61A			51.3	48.8
Secisbp2_T125A	57.1	41.6		
Psg25_*476fs		2.6	9.1	
Muc3_V326V		66.7		50.0
Cacna1h_C1449C	9.5		39.4	37.1
Ripply1_P100L	10.8		42.3	47.4
Tmem44_P254S	23.1		46.2	40.7
Fat1_A1844T	46.3	49.3	50.1	49.4
Frem3_S1580I	42.9	48.4	42.5	51.6
Zfpm2_E61G	56.9	37.5	52.7	48.3
Olfr1122_L44L	29.6	45.8	52.4	44.6
Olfr130_K185T	48.4	46.9	46.5	46.9
Olfr308_V78F	46.7	49.4	55.5	43.8
Gm5114_R545R	24.9	40.9	32.5	53.7
D430041D05Rik_T697M	43.4	52.6	60.6	60.0
Cbl_Y369delinsPLY			22.9	
Kras_G12S			36.7	
Flt3_D838E				94.9
Ptpn11_A465T			18.3	

Supplementary Figure 7. Full list of mutations in BM cells from mice injected with *CEBPA* N321D cells. Mice were culled 93 days post-injection ("donor cells") or, after transplantation of the donor BM cells into secondary recipients, either culled 4 months later (226 days intermediate) or monitored for the development of a leukaemia phenotype. Only mutations present in more than 2 samples per group are displayed. Each box shows the VAF of a mutation, with the blue colour intensity proportional to the VAF. Silent mutations are shown in green (no change in the coding amino acid). Mutations in RTK-RAS members are shown in red.

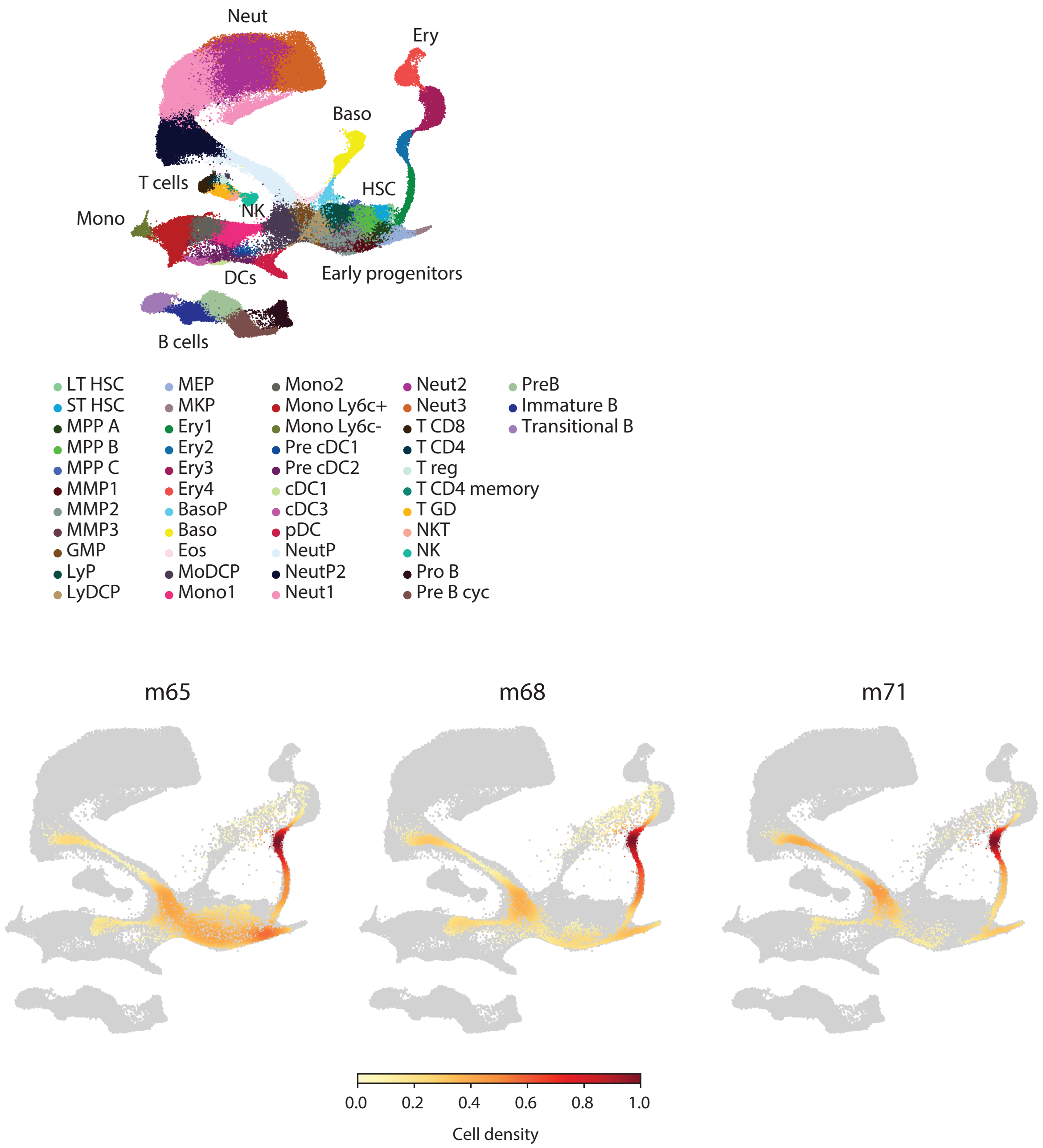

Supplementary Figure 8. Results of cell type annotation of Lineage⁻ cKit⁺ cells sorted from the m65, m68 and m71 donors at the 93-day time point.

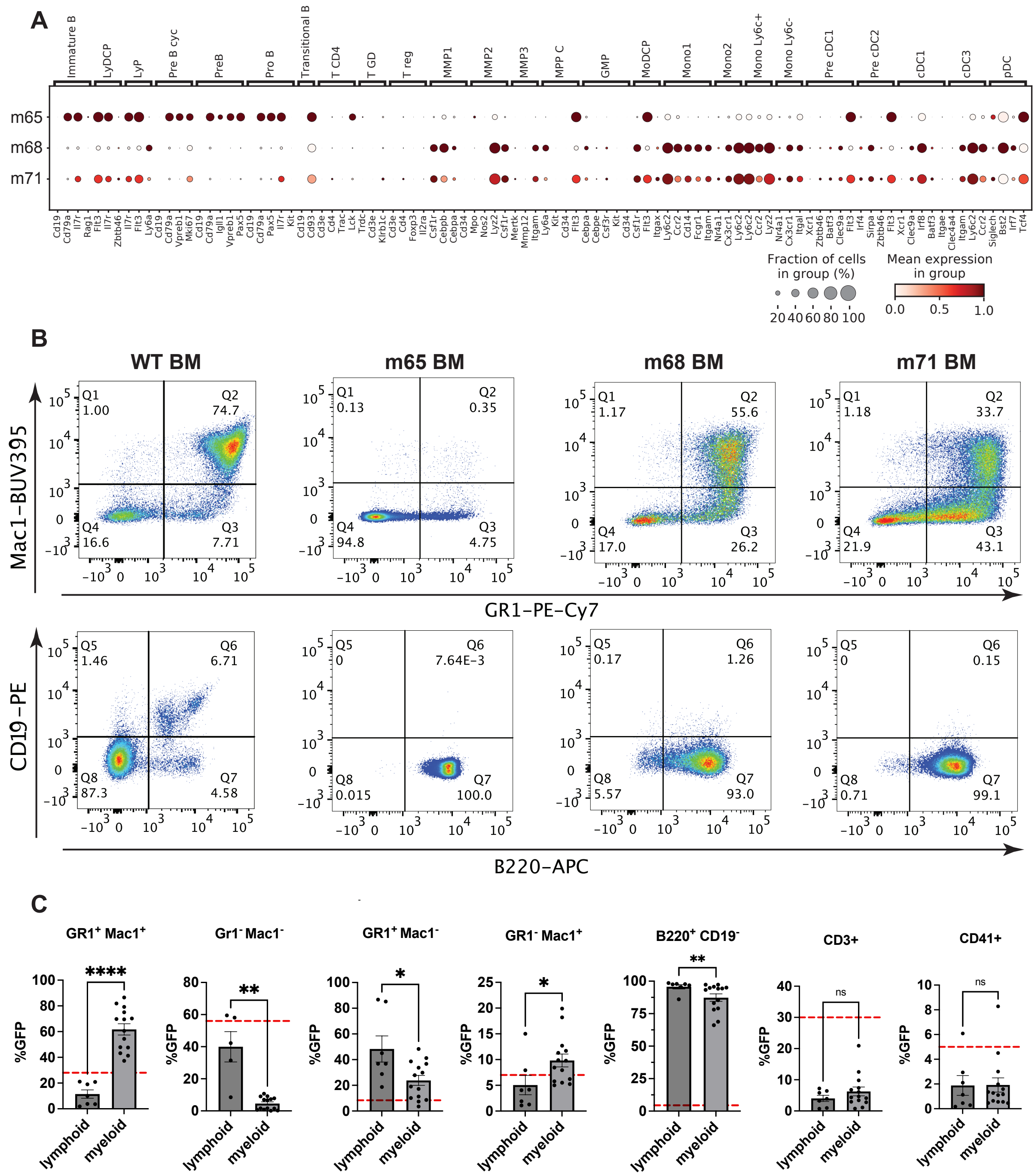

Supplementary Figure 9. Immunophenotypic characterisation of leukaemias derived from animals injected with *CEBPA* N231D cells. A. Dot plot showing transcriptional expression of myeloid and lymphoid lineage markers across annotated cell populations in the three tumours analysed by scRNA-seq. Marker expression is grouped by cell type, highlighting differences in lineage composition between tumours. **B.** Flow cytometric analysis of lineage distribution in bone marrow from a wild-type (WT) mouse and three representative tumours subjected to

scRNA-seq. Cells were assessed for myeloid (Mac-1, Gr-1) and lymphoid (B220, CD19) markers. Tumours segregate into distinct phenotypic groups, with one exhibiting a predominantly lymphoid profile and two displaying predominantly myeloid features.

C. Quantification of marker expression in spleen cells from all tumours generated in this study. Data are presented as the percentage of GFP-positive cells and grouped according to dominant phenotype (lymphoid-biased vs myeloid-biased tumours). The red dashed line indicates the expression of the respective markers in WT spleen acquired in the same analysis. Statistical significance was assessed using Mann–Whitney test (*p < 0.01, **p <0,001 , ***p<0.0001).

Supplementary Methods

Cell lines and culture conditions

Hoxb8-FL cell line was kindly provided by the Hans Häcker laboratory. Cells were grown at 37°C and 5% CO_2 in RPMI 1640 medium (Sigma R8758) supplemented with 10% Foetal Bovine Serum (FBS) (Gibco 10270-106), 5% Flt3L conditioned medium, 0.1% 2-Mercaptoethanol (Invitrogen 31350-010), 1% Penicillin/ Streptomycin (Sigma, P0781), 1% Glutamine (Sigma, G7513) and 1µM β -oestradiol (Sigma E2758). This media is termed as self-renewal media (Flt3L+Oe+) throughout the text. Cells were kept at densities between 0.7 - 12 x 10^5 cells/mL. Cells were regularly tested for mycoplasma by in house facility.

Flt3L conditioned medium was prepared using the B16-FLT3 cell line that constitutively expresses Flt3L and was kindly provided by Hans Häcker laboratory. The B16 cells were seeded in 10 cm dishes and grown at 37°C and 5% CO_2 in RPMI 1640 medium (Sigma R8758) supplemented with 10% FBS (Gibco 10270-106), 0.1% 2-Mercaptoethanol, 1% Penicillin/ Streptomycin (Sigma, P0781), 1% Glutamine (Sigma, G7513). Cells were cultured for 2-3 days

until confluent, the supernatant harvested, and media replaced daily over a 3-day period. The supernatant was then filtered, aliquoted and stored at -80°C.

293T cells were cultured in Dulbecco's Modified Eagle Medium (DMEM) supplemented with 10% FBS, 1% Penicillin/ Streptomycin (Sigma, P0781) and 1% Glutamine (Sigma, G7513). Cells were cultured in 100 mm/150 mm dishes at 37°C and 5% CO_2. Cells were passaged every 2-3 days with Trypsin-EDTA (Sigma, T3924) and kept at 30-90% confluency.

Differentiation media refers to the Flt3L media that induced differentiation of Hoxb8-FL cells and consisted of the same components that the Hoxb8-FL media except without the addition of β -oestradiol.

Cloning of the retroviral *CEBPA* N312D, *CEBPA* WT and Empty Vector (EV) plasmids

The pMSCV-IRES-eGFP (EV), pMSCV-WT (human) CEBPA – IRES – eGFP (CEBPA WT) and pMSCV – N321D (human) CEBPA – IRES – eGFP (*CEBPA* N321D) constructs were generated as follows.

The human CEBPA N321D construct was assembled from synthetic nucleotides (GeneArt Life Technologies) and inserted into a kanamycin-resistant vector to generate pMK-T CEBPA N321D. The sequence for the inserted human N321D version of the *CEBPA* gene is the following:

ATGGAGTCGGCCGACTTCTACGAGGCGGAGCCGCGGCCCCCGATGAGCAGCCAC

CTGCAGAGCCCCCCGCACGCGCCCAGCAGCGCCGCCTTCGGCTTTCCCCGGGGCG

CGGGCCCCGCGCAGCCTCCCGCCCCACCTGCCGCCCCGGAGCCGCTGGGCGGCA

TCTGCGAGCACGAGACGTCCATCGACATCAGCGCCTACATCGACCCGGCCGCCTT

CAACGACGAGTTCCTGGCCGACCTGTTCCAGCACAGCCGGCAGCAGGAGAAGGC
CAAGGCGGCCGTGGGCCCCACGGGCGGCGGCGGCGGCGGCGACTTTGACTACCC
GGGCGCGCCCGCGGGCCCCGGCGGCGCCGTCATGCCCGGGGGAGCGCACGGGCC
CCCGCCCGGCTACGGCTGCGCGGCCGCCGGCTACCTGGACGGCAGGCTGGAGCC
CCTGTACGAGCGCGTCGGGGCGCCGGCGCTGCGGCCGCTGGTGATCAAGCAGGA
GCCCCGCGAGGAGGATGAAGCCAAGCAGCTGGCGCTGGCCGGCCTCTTCCCTTA
CCAGCCGCCGCCGCCGCCGCCGCCCTCGCACCCGCACCCGCACCCGCCGCCCGC
GCACCTGGCCGCCCCGCACCTGCAGTTCCAGATCGCGCACTGCGGCCAGACCAC
CATGCACCTGCAGCCCGGTCACCCCACGCCGCCGCCCACGCCCGTGCCCAGCCCG
CACCCCGCGCCCGCGCTCGGTGCCGCCGGCCTGCCGGGCCCTGGCAGCGCGCTC
AAGGGGCTGGGCGCCGCGCACCCCGACCTCCGCGCGAGTGGCGGCAGCGGCGCG
GGCAAGGCCAAGAAGTCGGTGGACAAGAACAGCAACGAGTACCGGGTGCGGCG
CGAGCGCAACAACATCGCGGTGCGCAAGAGCCGCGACAAGGCCAAGCAGCGCA
ACGTGGAGACGCAGCAGAAGGTGCTGGAGCTGACCAGTGACGATGACCGCCTGC
GCAAGCGGGTGGAACAGCTGAGCCGCGAACTGGACACGCTGCGGGGCATCTTCC
GCCAGCTGCCAGAGAGCTCCTTGGTCAAGGCCATGGGCAACTGCGCGTGA

To generate the WT version of CEBPA, the pMK-T CEBPA N321D construct was digested with BlpI and BstAPI restriction enzymes (New England Biolabs) to excise a portion of CEBPA gene containing the mutated nucleotide. The resulting linearised product was run on an agarose gel and extracted with the QIAquick Gel Extraction Kit (Qiagen). Complementary primers containing the WT version of human CEBPA gene were annealed together and used to replace the region in the digested vector with the WT version of the gene.

To generate the retroviral constructs used in this study, the pMSCV-MLL-ENL-IRES-eGFP vector (obtained from S Basilico[1] was digested with Sal I and Bgl II restriction enzymes (New England Biolabs) to release the MLL-ENL fragment. The rest of the fragment was either re-ligated to generate the control pMSCV-IRES-eGFP (EV) plasmid or used to insert the CEBPA gene in the following manner. The CEBPA WT or CEBPA N321D cDNAs were first amplified by polymerase chain reaction (PCR) using KAPA HiFi HotStart ReadyMix (Kapa Biosystems, now Roche). PCR primers were designed to attach an in-frame FLAG tag and Sal I / Bgl II restriction sites to CEBPA inserts. The resulting CEBPA WT/N321D-Flag inserts were ligated into digested pMSCV-IRES-eGFP vector by Gibson assembly and transformed into electrocompetent DH-10β cells. Ampicillin-resistant colonies were selected by growth on agar plates with ampicillin. Plasmid DNA was isolated by an alkaline lysis method using buffers S1 (containing RNaseA), S2 and S3 from the NucleoBond®Xtra Maxi Kit (Macherey-Nagel). The resulting plasmids verified by Sanger Sequencing.

Retroviral production and transduction of Hoxb8-FL cells with *CEBPA* N321D, WT or EV

5 x 10^6 293T cells were seeded into 10 cm culture dishes approximately 16 hours before transfection, aiming for 80% cell confluency at the time of addition of the viral supernatant. 5 μg of DNA (MSCV-EV-GFP/MSCV-CEBPA-WT-GFP/MSCV-CEBPA-N321D-GFP) was mixed with 5 μg of Psi packaging vector, topped up to 10 μL with H_2O and added to 500 μL of plain DMEM (DNA/DMEM mix). In another tube, 500 μL of plain DMEM was mixed with 45 μL of TransIT-LT1 Transfection Reagent (Mirus MIR 2305, TransIT/DMEM mix). The DNA/DMEM mixture was slowly added to the TransIT/DMEM mix and incubated at room temperature for 30 mins. The resulting solution was slowly distributed in a drop wise fashion around the dish of 293T cells and cells incubated overnight at 37°C and 5% CO_2. 24 hours post

transfection, the 293T media in the dishes was replaced with 6 mL of self-renewal Hoxb8-FL cell media and cells were incubated at 37°C and 5% CO_2. After a further 24 hours, the cell supernatant was collected with a 5 mL syringe, taking extra care to not disturb the attached cells at the bottom of the dish. The collected viral supernatant was passed through a 0.45 µm filter to remove cell debris.

For transduction, 1.5 x 10^6 of Hoxb8-FL cells were placed in 2 mL of appropriate media in a well of a 6-well cell culture plate. 24 µL of 1 mg/mL polybrene (Sigma TR-1003-G) was added to the well to a final polybrene concentration of 8 µg/mL. 1 mL of viral supernatant (carrying either MSCV-EV-GFP, MSCV-CEBPA-WT-GFP, MSCV-CEBPA-N321D-GFP) was added to the well and gently mixed by pipetting up and down and swirling of the plate. Cells were centrifuged at 780 x g, at 32°C for 90 mins (Allegra 6R Rotor GH-3.8) with a "No break" setting. Following the spin, cells were incubated for 90 mins at 32°C and 5% CO_2. Subsequently, 1.5 mL of media was carefully removed from the side of the well (without disturbing the cells) and 2.5 mL of fresh media was added. Cells were incubated overnight at 37°C and 5% CO_2.

Differentiation conditions to generate the *CEBPA* N321D cells

Hoxb8-FL cells were transduced with either empty vector (EV), *CEBPA* WT or *CEBPA* N321D viral vectors and cultured for two days in self-renewing media (media used to culture the parental Hoxb8-FL cell line). Two days post transduction, cells were washed two times with phosphate buffered saline (PBS) (Sigma D8537) and resuspended in differentiation media (Flt3L) (RPMI 1640 medium (Sigma R8758) supplemented with 10% Foetal Bovine Serum (FBS) (Gibco 10270-106), 5% Flt3L conditioned medium, 0.1% 2-Mercaptoethanol (Invitrogen 31350-010), 1% Penicillin/ Streptomycin (Sigma, P0781) and 1% Glutamine

(Sigma, G7513)). Cells were counted using the CASY® Cell Counter, counting cells with diameter range of 7.85 µm - 14.95 µm. Cells were seeded at a concentration of 1 x 10^5 cells/mL in 6-well plates (2 mL total volume) and in T75 flasks (12 mL total volume) and cultured at 37°C and 5% CO_2. Medium was changed every 2-3 days, and the cells were kept at a concentration of 0.7 - 16 x 10^5 cells/mL. Differentiated cells that were adhered to the wells/flasks were dissociated enzymatically with Trypsin-EDTA (Sigma T3924) prior to processing. However, CEBPA N321D cells past Day 35 were not trypsinised as cells were no longer adherent. Presentation of haematopoietic markers was analysed by flow cytometry on the day cells were transferred into differentiation media (day 0) and at 5, 9, 14, 21 and 28 days after. The cells in plates were used for flow cytometry analysis at days 0, 5 and 9, the cells in flasks were used for all the subsequent flow cytometry characterisations and cell expansion. In the case of the CEBPA WT and EV cells, cells were monitored for 12 days until all cells differentiated and the rate of cell death precluded the continuation of the experiment. For CEBPA N321D cells, the cells were cultured for 35 days and cells frozen in 7 x 10^6 cells/mL aliquots in 1 mL of 10% DMSO/90% FBS solution.

Flow cytometry and sorting

Cells were centrifuged at 300 x g for 5 mins, washed two times with 2% FBS/PBS solution (Gibco 10270-106, Sigma D8537) and resuspended in 100 µl of 2% FBS/PBS. Cells were stained with an antibody mixture (see table below) and incubated in the dark at 4°C for 30 mins. Single stained controls were prepared using UltraComp eBeadsTM Compensation Beads (Thermo Fisher 01-2222-41). After incubation, cells were washed twice in 2% FBS/PBS, resuspended in 2% FBS/PBS and 1:175 dilution of 7-Aminoactinomycin D (7AAD) viability dye (BD 559925) was added to exclude dead cells. The LSR Fortessa (BD Biosciences) was used to analyse the cells. Antibodies used are listed below.

Antibody	Clone	Supplier	Catalogue	Dilution
c-kit (APC/Cy7)	2B8	BioLegend	105826	1:100 or 2.5:100
Ly-6G (BV510)	1A8	BioLegend	127633	1:100
B220 (AF700)	RA3-6B2	BioLegend	103232	1:100
Cd11b (BV711)	M1/70	BioLegend	101242	1:800
Cd11c (PE/Cy7)	N418	BioLegend	117318	1:100
SIRPα (PE)	P84	BD Biosciences	560107	1:100
XCR1 (APC)	ZET	BioLegend	148206	1:100
BST2 (BV421)	927	BD Horizon	566431	1:100
F4/80 (BUV395)	T45-2342	BD Horizon	565614	1:100
MHC Class II (PerCP/Cy5.5)	M5/114.15.2	BioLegend	107626	1:400
CD45.1 (PE/Cy7)	A20	BioLegend	110730	1:100
CD45.2 (APC/Cy7)	104	BioLegend	109824	1:100
CD45 (BV711)	30-F11	BioLegend	103147	1:100
CD3 (PB)	17A2	BioLegend	100214	2.5:100
CD41 (BV510)	MWReg30	BD Biosciences	740136	2.5:100
B220 (APC)	RA3-6B2	BioLegend	103212	2.5:100
Mac1 (BUV395)	M1/70	BD Biosciences	563553	2.5:100
Gr1 (PE/Cy7)	RB6-8C5	BioLegend	108416	2.5:100
CD19 (PE)	6D5	BioLegend	115508	2.5:100

In case of cell sorting, cells were sorted by the CIMR Flow Core Facility using the Influx Cell Sorter (BD Biosciences). Flow cytometry data was processed with the FlowJo software.

Microscopy

For morphological examination of EV and *CEBPA* N321D cells, cytospin preparations were carried out by loading 50–100 µL of cells into cytofunnels and centrifuging at 700 rpm for 2 minutes on a Shandon Cytospin 3. The resulting cytoslides were stained using a Rapid Romanowsky Stain Pack (TCS Biosciences, HS705) according to the manufacturer's protocol. Microscopic images were acquired on a Zeiss Axio Imager.Z2 using Zen Pro software.

Terminal blood samples were used to make blood smear slides, which were subsequently stained for haematoxylin followed by staining with eosin (H&E). Spleen, liver and bone marrow from symptomatic animals were preserved in formalin until submitted for processing and H&E staining to Cambridge Stem Cell Institute Histology facility. Images were taken on OLYMPUS VS200 Slideviewer.

Submission of mouse bone marrow cells for 10X Genomics Single Cell RNA-Sequencing

30,000-60,000 mouse bone marrow cells were FACS sorted for GFP+ 7-AAD- into 2% PBS/FBS solution. Cells were washed with 2% PBS/FBS solution and resuspended in 0.04% Bovine Serum Albumin (BSA, AMBION) solution at concentration of 371 cells/µL. 20,000 cells were submitted to the Cancer Research UK Cambridge Institute Single-Cell Facility for 10x Genomics single cell RNA-Sequencing using the Chromium Single Cell 3' Reagents (v3.1 Chemistry), libraries were prepared following manufacturers protocols and sequenced on an Illumina NovaSeq 6000.

DNA extraction and preparation of material for whole exome sequencing

DNA from bone marrow cells and the *CEBPA* N321D cells was extracted using the Qiagen DNeasy Blood and Tissue Kit according to the manufacturer's instructions for 'Purification of Total DNA from Animal Blood or Cells (Spin-Column Protocol)'. Around 2 - 5 x 10^6 cells were used for the extraction. The optional step of addition of 4 µl RNase A (100 mg/ml) was followed. DNA concentration was measured using the DeNovix Spectrophotometer. RNA contamination was checked by agarose gel electrophoresis. Exome capture and sequencing was performed by Dr. David Adams' laboratory at the Wellcome Trust Sanger Institute or by Novogene (UK). Whole exome sequencing was performed with the Illumina Novaseq6000 platform (Wellcome Trust Sanger Institute) or with the Novaseq X Plus Series (Novogene).

Sample processing for low-cell number RNA sequencing

Samples were processed using a modified version of the Smart-Seq2 protocol [2,3] described in Kucinski *et al* [4] with slight modifications. Briefly, cells were sorted into 96-well plates. For each well, 375 cells were sorted into 11.5 µl lysis buffer containing 1.15 µl SUPERase-In RNase Inhibitor (20 U/µl Thermo Fisher Scientific AM2696), 0.115 µl 100 mM DTT, 0.23 µl

10% Triton X-100 solution (Sigma 93443) and 10.005 µl Nuclease-free H_2O (Sigma W4502). Cells were vortexed and spun in a pre-cooled centrifuge for 2 minutes at 2000 rpm and stored at -80 °C.

Plates were thawed on ice and 5 µl of annealing mixture added (0.5 µl External RNA Controls Consortium (ERCC) RNA Spike-In (1:300,000 dilution, Thermo Fisher 4456740), 0.1 µl Oligo-dT 100 µM primer and 4.4 µl Nuclease-free H_2O (Sigma W4502)). Plates were centrifuged at 700 x g for 1 minute, incubated at 72°C for 3 minutes in a thermocycler and immediately cooled down on ice. 3.3 µl (or 1/5 of the total volume) of annealed material was transferred to a new plate and used for further processing. 6.7 µl of reverse transcription solution was added, containing 0.1 µl Maxima H Minus (200 U/µl, Thermo Fisher EP0753), 0.25 µl SUPERase-In RNase Inhibitor (20 U/µl Thermo Fisher Scientific AM2696), 1 µl dNTPs (10mM stock), 2 µl 5x Maxima RT buffer (Thermo Fisher EP0753), 0.2 µl TSO (100 µM, Qiagen), 1.875 µl PEG800 (40% v/v) (Sigma-Aldrich) and 1.275 µl Nuclease-free H_2O (Sigma W4502). Samples were incubated at 42°C for 90 minutes and followed by an incubation at 70°C for 15 minutes. The cDNA was amplified by PCR. 40 µl of PCR mix was added consisting of 1 µl Terra PCR Direct Polymerase (1.25 U/µl, Takara 639271), 25 µl 2x Terra PCR Direct Buffer, 1 µl ISPCR primer (10 µM) and 13 µl Nuclease-free H_2O (Sigma W4502). The PCR conditions were the following: 98°C for 3 mins (1 cycle); 98°C for 15 sec, 65°C for 30 sec, 68°C for 4 min (13 cycles); 72°C for 10 min (1 cycle). The PCR product was purified using AMPure XP beads (Beckman A63882).

Sequencing libraries were prepared as follows. 1.63 µl of Tagment DNA buffer, 0.60 µl of Tagmentase (Nextera XT DNA Library Preparation Kit, Illumina Fc-131-1096) and 1.03 µl cDNA (previously diluted to final concentration of 100-150 pg) were aliquoted into a new 96-

well PCR plate and incubated a 55°C for 10 mins in a thermocycler. Immediately after incubation 0.82 µl of 0.2% SDS was added to neutralise the reaction. 1.23 µl of N Index Primer, 1.23 µl of S Index Primer 2 (Illumina Nextera XT Index Kit v2 Sets A (FC-131-2001) and D (FC-131-2004)) and 4.97 µl of PCR Master Mix (2.30 µl Phusion HF Buffer (Thermo Fisher), 0.10 µl dNTP (25mM stock), 0.07 µl, Phusion Polymerase, 2.50 µl (2 U/µl Thermo Fisher F530L), Nuclease Free H_2O (Sigma W4502)) were added and PCR run with the following protocol: 72°C for 3 mins (1 cycle); 98°C for 15 sec, 65°C for 30 sec, 68°C for 4 min (13 cycles); 72°C for 10 min (1 cycle).

Library pool was cleaned with AMPure XP beads (Beckman A63882) and eluted in 35 µl EB (10mM Tris-Cl, pH 8.5; Qiagen 19086). Library size distribution was checked with High Sensitivity D5000 ScreenTape (Agilent 5067- 5592). Libraries were quantified with KAPA qPCR quantification kit (Roche 07960140001/KK4824) using the MX3000P qPCR System (Stratagene, now Agilent). The pooled library was sequenced using the Illumina HiSeq 4000.

Animals

Animal work was conducted using C57BL/6J mice. Animals were housed in pathogen-free animal facility in individually ventilated cages under a 12:12h light dark cycle, with food and water available ad libitum. Experiments were conducted under a UK Home Office project license (registration number: PP3042348), as per UK Home Office regulations. The animal work was performed in accordance with the Animals (Scientific Procedures) Act 1986, UK and was reviewed by the University of Cambridge Animal Welfare Ethical Review Body.

In vivo transplantations

Sample sizes were chosen based on prior experience with similar transplantation models and practical considerations. For all transplantation studies, 5 x 10^5 of CD45.1+ cells were mixed with 1 x 10^5 of CD45.1/2 helper cells and transplanted into lethally irradiated CD45.2 mice via tail vein injection. After transplantation, peripheral blood was withdrawn and chimerism was checked by measuring the levels of CD45.1, CD45.2 and green fluorescent protein (GFP) by flow cytometry analysis. Disease onset was also monitored by measuring white blood cells (WBC) using a Blood Cell Counter Machine (scil Vet abc PlusTM). Investigators were not blinded to group allocation during outcome assessment, including WBC measurement and flow cytometry analysis. Routine animal monitoring was performed by facility staff. The specifics for each *in vivo* experiment are outlined below.

(a) *Injection of Hoxb8-EV and CEBPA N321D cells into mouse recipients*

For primary tumour induction experiments, either EV or *CEBPA* N321D cells were used. Prior to transplantation, to ensure a homogeneous population of cells, EV cells were FACS sorted for GFP+ cells and grown in self-renewal (Flt3L+Oe+) medium whereas *CEBPA* N321D cells were confirmed to be 100 % GFP+ via flow cytometry and grown in Flt3L+ medium. 20 four-week-old female mice were injected, with 14 mice receiving the mutant *CEBPA* N321D cells and 3 mice transplanted with EV cells. One EV injected and two *CEBPA* N321D injected mice were excluded from the analysis due to early death caused by irradiation-related toxicity. All other animals were included. Randomisation: Animals were randomly picked to be either injected with *CEBPA* N321D or EV cells. As well randomly selected 4 animals out of the *CEBPA* N321D injected group for additional irradiation dosing.

(b) *Transplantation of primary tumours into secondary mouse recipients*

Two primary tumours which developed in mice from the experiment described in (a) were chosen. Non-sorted, bone marrow cells were injected into 4 (#76) and 5 (#79) mice per tumour. Both male and female recipient mice were used. Mice were between 8 - 14 weeks old.

(c) *Transplantation of cells for the Mutational Time course experiment (as shown in Figure 4)*

CEBPA N321D cells were injected into 8 nine-week-old female mouse recipients. Simultaneously, an aliquot of 60,000 CEBPA N321D cells was submitted for 10x Genomics single cell RNA-sequencing, 'pre-injection' timepoint. Instead of waiting for tumour development, 3 mice (cases) were culled 3 months after cells were injected and 20,000 cells from each case were submitted for 10x Genomics single cell RNA sequencing '3-months' timepoint. The remaining cells from each case were re-transplanted into new lethally irradiated recipients, with at least 3 mice injected with 500,000 total bone marrow cells per case. No control cells (EV), were injected in this round. From each of the three cases, mice were culled 4 months post-transplantation, '7-months' timepoint. Mice displaying signs of leukaemia development, including elevated white blood counts, were culled the bone marrow and spleen isolated, checked for percentage of GFP expression via flow cytometry and material frozen. Three samples of bone marrow cells from diseased mice were submitted for 10x Genomics single cell RNA-sequencing, where each of the three samples were recipients of one of the 3 cases from the '3-months' timepoint.

Statistical Analyses

Statistical analyses were performed using GraphPad Prism version 10.4.0. Comparisons between groups were performed using two-tailed Mann–Whitney tests. Survival analyses were conducted using the log-rank Mantel–Cox test. Sample sizes are indicated in figure legends.

Data distribution was not formally tested for normality; non-parametric tests were therefore used where appropriate. For small sample sizes, the variability within groups is presented as individual data points or summary statistics as indicated in figure legends.

Bioinformatics

Generation of a custom reference file

Unless otherwise stated, the following reference mouse genome file was used for the alignment of sequencing data. FASTA file containing the sequence information and gene annotation GTF file for Mus musculus GRCm38 (mm10) was downloaded from ENSEMBL version 93. Genome sequence and annotation were then augmented with the sequence details of the 92 ERCC RNA spike-in controls, pMSCV plasmid backbone, GFP and the portion of human *CEBPA* sequence with the N321D mutation used in the generation of the MSCV-CEBPA-N321D-GFP plasmid.

Analysis of 10x Genomics single-cell RNA-sequencing data

Read processing and alignment to the modified mm10 reference genome were performed using the 10x Genomics Cell Ranger Software Suite (version 7.0.1). Downstream analysis was performed with Scanpy framework[5]and functions from CellProject (https://github.com/Iwo-K/cellproject) with the singularity container from Kucinski *et al.*[6]. For quality control, cells with less than 500 genes or less than 5,000 counts were excluded. Genes not detected (zero counts across all cells) were removed. Potential doublets were detected using Scrublet[7] (expected doublet rate = 10%) and removed from the dataset. Data were normalised to a total of 10,000 counts per cell, followed by log-transformation (log1p).

To annotate cell types in the query dataset, a non-diseased bone marrow reference atlas was used in combination with functions from the CellProject package (as described in Kucinski *et al.* [6]). To reduce computational load, the reference was subsampled to a maximum of 1,000 cells per cell type. Log-normalised counts from the query and reference datasets were concatenated, and batch effects were removed using Seurat's CCA method[8] using only the highly variable genes from the reference. The reference data were scaled, and the first 50 principal components were computed. The corrected query values were then fit into the computed PCA space, and 15 nearest neighbours were identified between query and reference cells. These neighbours were used both to transfer cell labels (by majority vote) and to predict query coordinates in the reference PCA space, which were subsequently used to embed the query data into the reference UMAP space[9]. For visualization, a small subset of cells (760 out of 104,620) with a mitochondrial content greater than 7.5% was excluded. Additionally, only cells assigned to groups representing more than 2% of the total cells in each sample were retained. Finally, cell density in the reference UMAP space was calculated for each sample using Scanpy's [5] embedding_density function (scanpy.tl.embedding_density) and visualised.

Whole exome sequencing analysis

Forward and reverse reads were aligned with Burrow – Wheeler Aligner (BWA) mem algorithm (version 0.7.17) [10] using the custom reference mouse genome. The index for the reference file used for the alignment was created by BWA index [10] .For quality processing of aligned reads, tools available from the GATK (version 4.3.0) suite [11] were used. Aligned SAM files were sorted by coordinate with SortSam (Picard) tool. PCR duplicates were marked by MarkDuplicates (Picard). Next, the resulting files were processed to improve the accuracy of the sequencing assigned base quality scores. First, the recalibration table was calculated with the BaseRecalibrator tool (GATK) by supplying the Mouse Genomes Project SNP and indel

database (Release Version 5) as reference data for sites of known variation. Secondly, the resulting table was used to recalibrate the base quality scores of the input reads with the ApplyBQSR tool (GATK). The recalibrated files were used to detect short somatic variants with Mutect2 (GATK). Detected mutations include single nucleotide changes, small insertions and deletions. The analysis was run for each tumour sample against a matched normal, where the normal sample was the preinjected *CEBPA* N321D cell line. Low quality variants were flagged using FilterMutectCalls (GATK) with default parameters. Variants that passed the filtering were filtered into a new file with BCFtools (1.9) view command[12] . Variant annotation was done using the ANNOVAR package[13]. Mouse gene definition database was created for the mm10 mouse genome following ANNOVAR's documentation by first downloading the UCSC mm10 annotation database via annotate_variation.pl -downdb and then generating a corresponding FASTA file with annotate_variation.pl –buildver and re-trieve_seq_from_fasta.pl generating a FASTA file with 47,382 mouse mm10 genomic regions. To generate a gene-based annotation the VCF files containing unannotated variant regions (from the 'Variant calling' step) were converted to annovar objects with convert2annovar.pl with -allsample –include –comment arguments. The resulting files were used to annotate the variants to genes via annotate_variation.pl –geneanno and default parameters. The resulting annovar objects was converted to maf files with the maftools package (version 2.12.00) [14] and visualisations created with maftools in R and custom code in Python.

Analysis of low-cell number Smart-Seq2 RNA sequencing data

Sequencing reads were aligned to a modified mm10 reference genome (described above) using STAR aligner, and gene counts were quantified with featureCounts. Further analysis was performed using the in-house smqpp package (https://github.com/SharonWang/smqpp). Cells were filtered out if they met any of the following criteria: fewer than 100,000 reads; fewer than

4,000 highly abundant genes (detected above 10 counts per million); fraction of gene-derived counts relative to total counts below 15%; more than 12% ERCC-derived counts; or more than 5% mitochondrial-derived counts. Genes detected in fewer than 1 cell were filtered out and remaining counts were normalised using the smqpp.normalise_data function based on DESeq2[15] size factor normalisation. The data were log-transformed, and 1,131 highly variable genes were identified. The data were then scaled, and PCA was performed on the expression values of the highly variable genes, retaining 50 principal components. A k-nearest neighbour graph was computed using 15 neighbours and the first 40 principal components, and UMAP embedding was generated.

References

1 Basilico S, Wang X, Kennedy A, Tzelepis K, Giotopoulos G, Kinston SJ *et al.* Dissecting the early steps of MLL induced leukaemogenic transformation using a mouse model of AML. *Nat Commun* 2020; 11. doi:10.1038/s41467-020-15220-0.

2 Picelli S, Faridani OR, Björklund ÅK, Winberg G, Sagasser S, Sandberg R. Full-length RNA-seq from single cells using Smart-seq2. *Nat Protoc* 2014; 9. doi:10.1038/nprot.2014.006.

3 Bagnoli JW, Ziegenhain C, Janjic A, Wange LE, Vieth B, Parekh S *et al.* Sensitive and powerful single-cell RNA sequencing using mcSCRB-seq. *Nat Commun* 2018; 9. doi:10.1038/s41467-018-05347-6.

4 Kucinski I, Wilson NK, Hannah R, Kinston SJ, Cauchy P, Lenaerts A *et al.* Interactions between lineage-associated transcription factors govern haematopoietic progenitor states. *EMBO J* 2020; 39. doi:10.15252/embj.2020104983.

5 Wolf FA, Angerer P, Theis FJ. SCANPY: Large-scale single-cell gene expression data analysis. *Genome Biol* 2018; 19. doi:10.1186/s13059-017-1382-0.

6 Kucinski I, Campos J, Barile M, Severi F, Bohin N, Moreira PN *et al.* A time- and single-cell-resolved model of murine bone marrow hematopoiesis. *Cell Stem Cell* 2024; 31. doi:10.1016/j.stem.2023.12.001.

7 Wolock SL, Lopez R, Klein AM. Scrublet: Computational Identification of Cell Doublets in Single-Cell Transcriptomic Data. *Cell Syst* 2019; 8. doi:10.1016/j.cels.2018.11.005.

8 Stuart T, Butler A, Hoffman P, Hafemeister C, Papalexi E, Mauck WM *et al.* Comprehensive Integration of Single-Cell Data. *Cell* 2019; 177. doi:10.1016/j.cell.2019.05.031.

9 McInnes L, Healy J, Melville J. UMAP: Uniform Manifold Approximation and Projection for Dimension Reduction. *ArXiv* 2020.http://arxiv.org/abs/1802.03426.

10 Li H. Aligning sequence reads, clone sequences and assembly contigs with BWA-MEM. 2013.http://arxiv.org/abs/1303.3997.

11 Auwera G van der, O'Connor BD. *Genomics in the cloud : using Docker, GATK, and WDL in Terra*. First edition. O'Reilly Media: Sebastopol, CA, 2020.
12 Danecek P, Bonfield JK, Liddle J, Marshall J, Ohan V, Pollard MO *et al.* Twelve years of SAMtools and BCFtools. *Gigascience* 2021; 10. doi:10.1093/gigascience/giab008.
13 Wang K, Li M, Hakonarson H. ANNOVAR: Functional annotation of genetic variants from high-throughput sequencing data. *Nucleic Acids Res* 2010; 38. doi:10.1093/nar/gkq603.
14 Mayakonda A, Lin DC, Assenov Y, Plass C, Koeffler HP. Maftools: Efficient and comprehensive analysis of somatic variants in cancer. *Genome Res* 2018; 28. doi:10.1101/gr.239244.118.
15 Love MI, Huber W, Anders S. Moderated estimation of fold change and dispersion for RNA-seq data with DESeq2. *Genome Biol* 2014; 15. doi:10.1186/s13059-014-0550-8.
